# Botanical Medicines Cryptolepis sanguinolenta, Artemisia annua, Scutellaria baicalensis, Polygonum cuspidatum, and Alchornea cordifolia Demonstrate Inhibitory Activity Against Babesia duncani

**DOI:** 10.3389/fcimb.2021.624745

**Published:** 2021-03-08

**Authors:** Yumin Zhang, Hector Alvarez-Manzo, Jacob Leone, Sunjya Schweig, Ying Zhang

**Affiliations:** ^1^ Department of Molecular Microbiology and Immunology, Johns Hopkins Bloomberg School of Public Health, Johns Hopkins University, Baltimore, MD, United States; ^2^ FOCUS Health Group, Naturopathic, Novato, CA, United States; ^3^ California Center for Functional Medicine, Kensington, CA, United States; ^4^ State Key Laboratory for the Diagnosis and Treatment of Infectious Diseases, National Clinical Research Center for Infectious Diseases, The First Affiliated Hospital, Zhejiang University School of Medicine, Hangzhou, China

**Keywords:** *Babesia duncani*, *Cryptolepis sanguinolenta*, herbal medicine, cryptolepine, artemisinin, baicalein, phytonutrient, phytotherapy

## Abstract

Human babesiosis is a CDC reportable disease in the United States and is recognized as an emerging health risk in multiple parts of the world. The current treatment for human babesiosis is suboptimal due to treatment failures and unwanted side effects. Although *Babesia duncani* was first described almost 30 years ago, further research is needed to elucidate its pathogenesis and clarify optimal treatment regimens. Here, we screened a panel of herbal medicines and identified *Cryptolepis sanguinolenta*, *Artemisia annua*, *Scutellaria baicalensis, Alchornea cordifolia*, and *Polygonum cuspidatum* to have good *in vitro* inhibitory activity against *B. duncani* in the hamster erythrocyte model. Furthermore, we found their potential bioactive compounds, cryptolepine, artemisinin, artesunate, artemether, and baicalein, to have good activity against *B. duncani*, with IC_50_ values of 3.4 μM, 14 μM, 7.4 μM, 7.8 μM, and 12 μM, respectively, which are comparable or lower than that of the currently used drugs quinine (10 μM) and clindamycin (37 μM). *B. duncani* treated with cryptolepine and quinine at their respective 1×, 2×, 4× and 8× IC_50_ values, and by artemether at 8× IC_50_ for three days could not regrow in subculture. Additionally, *Cryptolepis sanguinolenta* 90% ethanol extract also exhibited no regrowth after 6 days of subculture at doses of 2×, 4×, and 8× IC_50_ values. Our results indicate that some botanical medicines and their active constituents have potent activity against *B. duncani in vitro* and may be further explored for more effective treatment of babesiosis.

## Introduction

Babesiosis is a disease caused by the *Babesia* parasite that infects red blood cells. *Babesia* genus belongs to the apicomplexan phylum, and over 100 *Babesia* species have been identified (Birkenheuer et al., 2006). However, only a few of these have been documented to infect humans. *Babesia divergens*, *Babesia microti*, and *Babesia duncani* cause the most human babesiosis cases worldwide. Most human babesiosis infections in the United States are caused by *B. microti* and *B. duncani*, and in Europe the majority of reported cases are due to *B. divergens* (Vannier and Krause, 2012). All of these *Babesia* species can be acquired by *Ixodes* ticks that are also reservoirs of *Borrelia* spirochetes which cause Lyme disease. Recently, *Dermacentor albipictus* has been implicated as a competent tick vector for *Babesia duncani* (Swei et al., 2019). Epidemiologic studies have documented that up to 23% of patients with babesiosis also experienced concurrent Lyme disease (Diuk-Wasser et al., 2016) and coinfected patients can experience a greater number of symptoms for a longer duration than those with Lyme disease alone (Krause et al., 1996; Krause et al., 2002). In addition to transmission by tick bite, human babesiosis transmission has also been reported *via* blood transfusion, organ transplantation, and vertical transmission during pregnancy (Lux et al., 2003; Leiby, 2006). The clinical manifestations of babesiosis range from asymptomatic to severe and common clinical symptoms in patients include fever, chills, and sweats. Patients with immunological diseases, on immunosuppressive therapies, and those who have undergone splenectomy are at increased risk of more severe symptoms and even death (Vannier et al., 2015).

Babesiosis caused by *Babesia microti* is endemic in the Northeast and the upper Midwest of the United States. Babesiosis caused by *B. duncani* was first described in Washington State as strain WA1 in 1991 and is widespread in North America based on molecular and immunological tests (Vannier and Krause, 2012; Scott and Scott, 2018). Animals such as mice, gerbils, and hamsters are susceptible to infection with *B. duncani via* intraperitoneal injection. Studies in animal models show that *B. duncani* has a different pathogenesis than *B. microti*, in that the parasitemia in hamsters infected with *B. microti* can initially be very high, but then decrease to undetectable levels within several weeks. In contrast, *B. duncani* can cause severe acute disease and death in hamsters, within 10 days after inoculation (Kjemtrup and Conrad, 2000). In mouse models, *B. duncani* can give rise to more severe presentations and higher mortality rates than *B. microti* (Moro et al., 1998). In terms of morphology, there is no obvious difference in intraerythrocytic stages between *B. duncani* and *B. microti*, nevertheless, based on the phylogenetic analysis of 18S RNA gene, *ITS2* gene, *cytb* gene, and *coxI* gene, *B. duncani* show a distinct evolutionary lineage compared to *B. microti* and other *Babesia* species (Virji et al., 2019).

The current treatment protocols for human babesiosis frequently use medications such as atovaquone, azithromycin, clindamycin, quinine, and their combinations (Smith et al., 2020). However, these regimens are suboptimal and are associated with treatment failures and significant side effects (Kjemtrup and Conrad, 2000), even in immunocompetent patients (Krause et al., 1998; Gonzalez et al., 2014). Some cases of *Babesia* infection can be persistent, and recrudescence may occur up to two years after therapy (Krause et al., 2008; Raffalli and Wormser, 2016). Furthermore, it has been demonstrated that *B. duncani* showed unexpectedly high tolerance to atovaquone, azithromycin, clindamycin, and quinine *in vitro* (Abraham et al., 2018), and *B. duncani* could regrow *in vitro* after exposure to high concentrations of atovaquone or azithromycin for 3 days (Zhang et al., 2020). Therefore, alternative more effective antimicrobial agents and treatment regimens with fewer side effects need to be developed.

In the past decade, some promising anti-parasite drug candidates or compounds have been identified against *B. microti*, *B. divergens*, *B. gibsoni*, *B. bovis*, *B. bigemina*, and *B. caballi in vitro* or *in vivo* including mycophenolic acid, pentamidine, doxorubicin hydrochloride, vorinostat, luteolin, pyronaridine, robenidine, primaquine, and diphenyl furan for potential medical and veterinary treatment (Nehrbass-Stuedli et al., 2011; Rizk et al., 2015; Yao et al., 2015; Rizk et al., 2018; Li et al., 2020; Rizk et al., 2020). However, to date, limited research has focused on drug screening against *B. duncani*.

Herbal medicines were used by ancient cultures and their safety and efficacy have been documented by various traditional medicine systems such as Ayurveda and Traditional Chinese Medicine (Borchardt, 2002; Jaiswal et al., 2016). The adverse effects of botanical products were determined to be rare according to a recent report (Di Lorenzo et al., 2015). In a previous study, we have screened a panel of essential oils and found that garlic oil, black pepper, and their constituents showed good activity against *B. duncani* in a hamster erythrocyte model (Zhang et al., 2020). These findings indicated that natural products extracted from plants and herbs may serve as a potential source of promising compounds for anti-parasitic drugs. In this current study, we used the same hamster erythrocyte model to screen for inhibition of *B. duncani* using a panel of 46 herbal medicine extracts, many of which are useful in the treatment of babesiosis and related conditions according to the Buhner book on herbal medicines (Buhner, 2015) or were shown in our previous *in vitro* studies to have antimicrobial effects (Feng J. et al., 2020). Our results identified *Cryptolepis sanguinolenta, Artemisia annua, Scutellaria baicalensis, Alchornea cordifolia*, and *Polygonum cuspidatum* and their bioactive compounds as having good activity against *B. duncani*.

## Materials and Methods

### Hamster Donor Blood and *Babesia duncani* Culture

Hamster whole blood was collected from Golden Syrian hamsters (Charles River) by cardiac puncture using phosphate buffer saline containing 15 mM EDTA as anticoagulant according to protocols approved by the Johns Hopkins Institutional Animal Care and Use Committee. Hamster whole Blood was washed three times by centrifugation at 500 g for 15 min in PBS, 15mM EDTA solution with careful removal of the supernatant and buffy coat at each wash. After the last wash, red blood cells (RBCs) were resuspended at a concentration of 50% hematocrit in Puck’s saline glucose buffer with extra glucose (20 g glucose/L) and stored at 4°C less than 2 weeks before use. *Babesia duncani* strain WA1 (ATCC PRA-302) was cultured *in vitro* in HaRGM medium with fresh hamster erythrocytes at 37°C under an atmosphere of 5% CO_2_ with 95% humidity. Hamster erythrocytes were prepared with HaRGM medium, and continuous *B. duncani* culture method was performed as described in detail in our previous publication (Zhang et al., 2020).

### Natural Products and Compounds

A panel of 46 herbal medicine extracts (**Table 1**) and relevant solvent controls were used for testing. The herbal medicine extracts of *Uncaria tomentosa*, *Stevia rebaudiana*, *Juglans nigra*, *Cryptolepis sanguinolenta*, *Artemisia annua*, *Andrographis paniculata*, *Dipsacus fullonum*, *Withania somnifera*, *Scutellaria baicalensis*, and *Polygonum cuspidatum* were sourced from KW Botanicals (San Anselmo, California) and Heron Botanicals (Kingston, Washington). Most of these natural products were provided as ethanol extracts at 30%, 60%, and 90% ethanol, and the ethanol solvent was also tested as a control in the respective concentrations. Other herbal medicine extracts were purchased commercially. Detailed information including source, extraction parts, extraction type, and manufacturer of all the herbal medicine extracts tested in this study is shown in **Supplementary Table S1**. Cryptolepine (cryptolepine hydrate; Sigma-Aldrich), artemisinin (Alfa Aesar), artesunate (TCI Chemicals), artemether (Frontier Scientific), baicalein (5,6,7-Trihydroxyflavone; Alfa Aesar), quinine (quinine hydrochloride; Frontier Scientific), and clindamycin (clindamycin hydrochloride; Sigma-Aldrich) were prepared in DMSO stock in high concentrations and then diluted to desired concentrations with DMSO.

**Table 1:** Evaluation of a panel of 46 herbal medicines at 0.01% (v/v) for inhibitory activity against *B. duncani* after 3 days of incubation.

Product Names	*Plants*	Inhibition (%)
**Chinese Skullcap (90% EE)**	*Scutellaria baicalensis*	84
**Cryptolepis (90% EE)**	*Cryptolepis sanguinolenta*	80
**Cryptolepis (60% EE)**	*Cryptolepis sanguinolenta*	70
**Chinese Skullcap (60% EE)**	*Scutellaria baicalensis*	68
**Japanese knotweed (60% EE)**	*Polygonum cuspidatum*	59
**Sweet wormwood (30% EE)**	*Artemisia annua*	58
**Alchornea**	*Alchornea cordifolia*	54
**Japanese knotweed (90% EE)**	*Polygonum cuspidatum*	42
Andrographis (90% EE)	*Andrographis paniculata*	37
Andrographis (60% EE)	*Andrographis paniculata*	36
Sweet wormwood (60% EE)	*Artemisia annua*	35
Andrographis (30% EE)	*Andrographis paniculata*	34
Cistus	*Cistus incanus*	34
Ashwagandha (30% EE)	*Withania somnifera*	33
Hemp oil	*Cannabis sativa*	26
Barberry	*Berberis vulgaris*	25
Chuan xin lian	*Andrographis paniculata*	23
Black walnut (90% EE)	*Juglans nigra*	23
Ashwagandha (60% EE)	*Withania somnifera*	23
Stevia/Tian ju ye	*Stevia rebaudiana fol*	22
Ashwagandha (90% EE)	*Withania somnifera*	21
Echinacea	*Echinacea purpurea* & *Echinacea angustifolia*	20
Andrographis	*Andrographis paniculata*	19
Licorice	*Glycyrrhiza* spp.	19
Grapefruit seed extract	*Grapefruit seed*	17
Usnea	*Usnea* spp.	17
Eleuthero	*Eleutherococcus senticosus*	17
Black walnut (60% EE)	*Juglans nigra*	13
Sweet wormwood (90% EE)	*Artemisia annua*	13
Gou teng	*Uncaria rhynchophylla*	12
Teasel/Gao liang jiang	*Dipsacus fullonum*	11
Osha	*Ligusticum porter root*	11
Cryptolepis (30% EE)	*Cryptolepis sanguinolenta*	9
Chinese Skullcap (30% EE)	*Scutellaria baicalensis*	9
Houttuynia	*Houttuynia*	9
Bidens	*Bidens pilosa*	9
Ban zhi lian	*Scutellaria barbata*	8
Black walnut (30% EE)	*Juglans nigra*	8
Uncaria	*Uncaria tomentosa*	6
Samento	*Uncaria tomentosa*	0
Cumanda	*Campsiandra angustifolia*	0
Banderol	*Otoba* sp.	0
Coptis	*Rhizoma coptidis*	0
Japanese knotweed (30% EE)	*Polygonum cuspidatum*	0
Reishi	*Ganoderma linghzi*	0
Black Walnut	*Juglans nigra fruc*	0

The growth of infected erythrocytes at day zero was set as 0%. The growth of B. duncani in infected erythrocytes treated with 1% DMSO vehicle at day three was set as 100%. EE, ethanol extract. The bold product names indicate the effective hits studied in this study.

### *In Vitro* Evaluation of Herbal Medicines and Compounds on Inhibition of *B. duncani*

*Babesia duncani* was cultured in 24-well plate at 2.5% hematocrit of hamster erythrocytes at 37°C under an atmosphere of 5% CO_2_ with 95% humidity for 3–4 days. When parasitemia reached 2%, the infected erythrocytes were split evenly into 96-well plates at 2.5% hematocrit each well for natural products and compounds test. Natural products were prepared in 10% (v/v) DMSO stock solution. Ethanol at 30%, 60%, and 90% was prepared in parallel in DMSO as controls. Then the natural products and ethanol controls were added to 96-well plates containing infected erythrocytes to obtain final concentrations of 0.01%. Treated cultures were inoculated at 37°C for 3 days without replacing medium in a chamber under an atmosphere of 5% CO_2_ with 95% humidity. Then 1 µl of erythrocytes laid on the plate bottom were taken out for growth test by SYBR Green I assay as described previously (Zhang et al., 2020). Briefly, the 1 μl of RBCs sediment were placed into 100 μl of lysis buffer consisting of 20 mM Tris, pH 7.4, 5 mM EDTA, 0.008% saponin, 0.08% Triton X-100, and 2× SYBR Green I (10,000× stock, Invitrogen) in 96-well plates. The plates were then inoculated in the dark at 37°C for 60 mins followed by plate reading at excitation wavelength (490 nm) and a fluorescence intensity at 520 nm in a microplate reader (HTS 7000 plus Bio Assay Reader, PerkinElmer Inc., USA). For the IC_50_ susceptibility assay, *B. duncani* cultures were exposed to increasing concentrations of *Artemisia annua* (30% ethanol extract), *Cryptolepis sanguinolenta* (90% ethanol extract), *Scutellaria baicalensis* (90% ethanol extract), cryptolepine, artemisinin, artesunate, artemether, baicalein, quinine, and clindamycin. Each IC_50_ was performed in triplicate in 96-well plates with a starting 2% parasitemia and 2.5% hematocrit followed by SYBR Green I stain. Dose-response curves with fitting a nonlinear regression curve were performed in GraphPad Prism (version 7.0) and then calculate the best-fit IC_50_ values. The morphology of Giemsa stained thin blood smears of *B. duncani* after treatment with compounds at 1×, 2×, and 4× IC_50_ concentrations for 3 days was observed under BZ-X710 All-in-One Fluorescence Microscope (KEYENCE, Inc., Itasca, IL, USA).

### Subculture Studies to Evaluate Killing Efficacy of the Top Natural Product Hits and Selected Compounds

*B. duncani* cultures were exposed to *Artemisia* annua (30% ethanol extract), *Cryptolepis sanguinolenta* (90% ethanol extract), *Scutellaria baicalensis* (90% ethanol extract), cryptolepine, artemisinin, artesunate, artemether, baicalein, quinine, and clindamycin at their respective 1×, 2×, 4× and 8× IC_50_ values for 3 days. After three days of exposure, the treated erythrocytes were washed three times in HaRGM medium to wash away drugs and then inoculated in fresh hamster erythrocytes at ratio of 1:5. Each compound was tested in triplicate, and every well had 4–6 replicates for sampling to avoid obvious hematocrit decrease. Culture conditions of the subcultures were identical to those described above. One microliter of erythrocytes laid on the plate bottom was taken out every one or two days and stored at -80°C. The growth of the subculture was examined by the SYBR Green I assay as described above, and the fluorescence units of day zero were normalized as zero.

## Results

### Evaluation of Natural Product Extracts for Inhibitory Activity Against *B. duncani*

We evaluated a panel of 46 herbal medicine extracts and their corresponding controls at a concentration of 0.01% (v/v) for inhibitory activity against *B. duncani* at an initial parasitemia of 2% *in vitro* after 3 days of incubation (**Table 1**). *Cryptolepis sanguinolenta* (60, 90% ethanol extracts), *Artemisia annua* (30% ethanol extract), *Scutellaria baicalensis* (60%, 90% ethanol extracts), *Polygonum cuspidatum* (60% ethanol extract), and *Alchornea cordifolia* were the top hits with more than 50% inhibitory effect at 0.01% against *B. duncani*. As a control, we tested the ethanol carrier at concentrations of 30%, 60%, and 90%, which did not show obvious inhibitory effect at up to 1% concentration.

**Figure 1:**
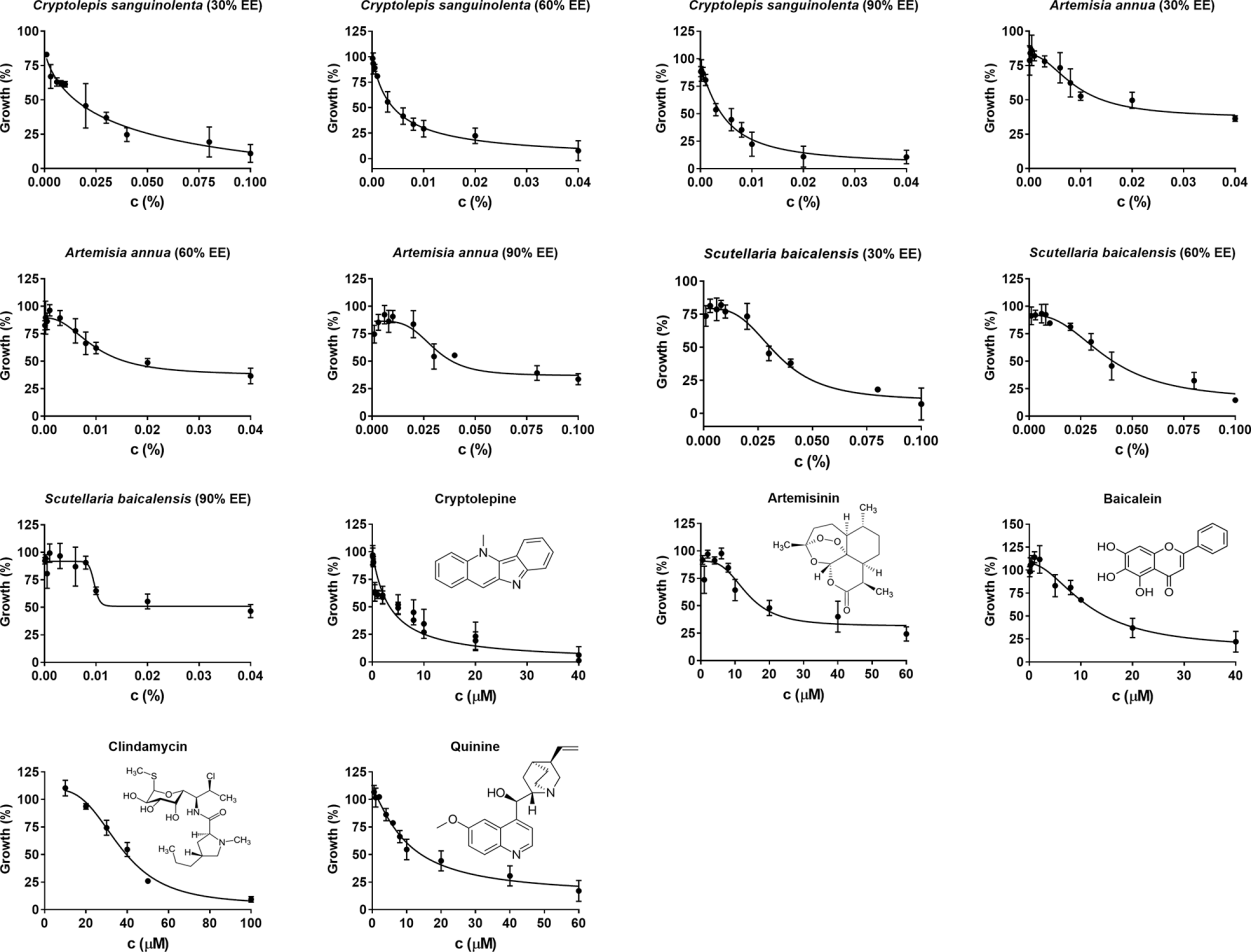
Evaluation of *in vitro* natural products and drug compounds’ susceptibility of *B. duncani* to *Cryptolepis sanguilonlenta* (30%, 60%, and 90% EE), *Artemisia annua* (30%, 60%, and 90% EE), *Scutellaria baicalensis* (30%, 60%, and 90% EE), cryptolepine, artesunate, baicalein, clindamycin, and quinine at different concentrations. Each natural product or drug compound concentration was set in triplicate. SYBR Green I assay was performed at day three after exposure to the natural products or drug compounds. The growth of *B. duncani* in infected erythrocytes at day zero was set as 0%. The growth of *B. duncani* in infected erythrocytes treated with 1% DMSO vehicle at day three was set as 100%. GraphPad Prism (version 7.0) was used to generate dose-response curves by fitting a nonlinear regression curve to the data. c, concentration; EE, ethanol extract.

*Cryptolepis sanguinolenta*, *Artemisia annua*, and *Scutellaria baicalensis* showed activity at 30%, 60%, and 90% EE, therefore, we further tested the half-maximal inhibitory concentration values of these three active natural product extracts at different ethanol concentrations. Dose-response assays confirmed that *Cryptolepis sanguinolenta* and *Scutellaria baicalensis* at 60% and 90% EE showed approximately 4–10 times lower IC_50_ values compared to those at 30% EE. In contrast, *Artemisia annua* at 30% EE exhibited the lowest IC_50_ value, even lower than it was at 60% and 90% EE (**Table 2**; **Figure 1**).

**Table 2:** The differing IC_50_ values indicated that *Cryptolepis sanguinolenta*, *Artemisia annua*, and *Scutellaria baicalensis* showed different inhibitory effects in 30%, 60%, and 90% ethanol extracts.

Natural product extracts	IC_50_ values
*Cryptolepis sanguinolenta* (30% EE)	0.039% (v/v)
*Cryptolepis sanguinolenta* (60% EE)	0.0041%
*Cryptolepis sanguinolenta* (90% EE)	0.0046%
*Artemisia annua* (30% EE)	0.0091%
*Artemisia annua* (60% EE)	0.0097%
*Artemisia annua* (90% EE)	0.030%
*Scutellaria baicalensis* (30% EE)	0.034%
*Scutellaria baicalensis* (60% EE)	0.038%
*Scutellaria baicalensis* (90% EE)	0.0097%
Cryptolepine	3.4 µM
Artemisinin	14 µM
Baicalein	12 µM
Quinine	10 µM
Clindamycin	37 µM

Growth was evaluated by SYBR Green stain at day three after B. duncani exposure to natural product extracts. Each natural product concentration was made in triplicate. IC_50_ values were calculated in GraphPad Prism (version 7.0). EE, ethanol extract.

### Artemisinin, Cryptolepine, and Baicalein Are the Active Constituents Extracted From *Artemisia annua, Cryptolepis sanguinolenta*, and *Scutellaria baicalensis* Respectively and Have High Potent Antibabesial Activity

Since *Cryptolepis sanguinolenta* (90% ethanol extract), *Artemisia annua* (30% ethanol extract), and *Scutellaria baicalensis* (90% ethanol extract) extracts showed better inhibitory activity against *B. duncani* than *Polygonum cuspidatum*, and *Alchornea cordifolia*, we focused on testing cryptolepine, artemisinin, and baicalein, which are known bioactive compounds derived from these three herbal medicines (Klayman, 1993; Cimanga et al., 1996; Nishioka et al., 1998). For comparison, we also tested the commonly used clinical drugs quinine and clindamycin. All these compounds and drugs inhibited the growth of *B. duncani* in a dose-dependent manner (**Figure 1**). The IC_50_ values of cryptolepine, artemisinin, and baicalein were 3.4 μM, 14 μM, and 12 μM, respectively. In contrast, the IC_50_ values of quinine and clindamycin were 10 μM and 37 μM.

### Artemisinin Derivatives Artesunate and Artemether Showed Higher Inhibitory Activity Against *B. duncani* Than Artemisinin

**Figure 2:**
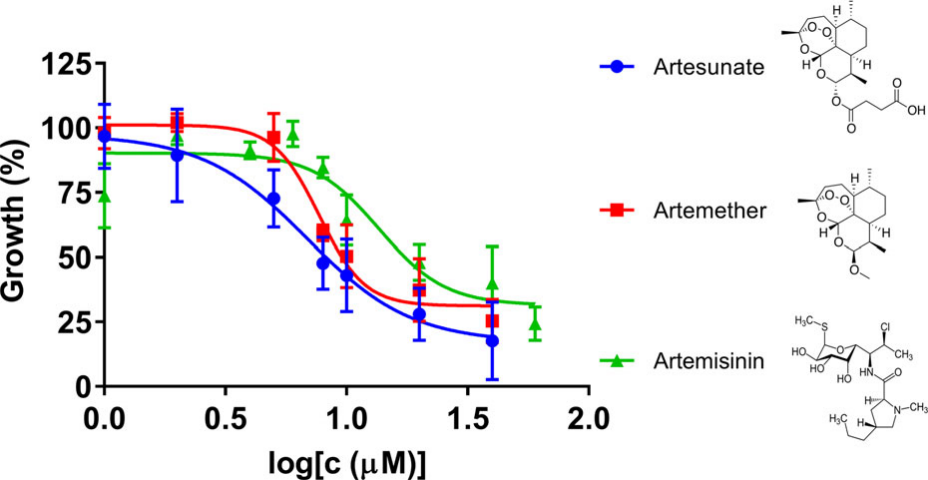
The *in vitro* inhibitory activity of artesunate, artemether, and artemisinin against *B. duncani* at an initial parasitemia of 2% after three days incubation. The growth of *B. duncani* in infected erythrocytes at day zero was set as 0%, and in the infected erythrocytes treated with 1% DMSO vehicle at day three was set as 100%.

Artemisinin derivatives, including artesunate and artemether, are potent antimalarial drugs which have been shown to reduce malaria parasitemia more rapidly than any other known antimalarial drugs, and are effective against multidrug-resistant malaria parasites (Olliaro et al., 2001). In our research, we compared artemisinin, artesunate, and artemether for their inhibitory effects against *B. duncani* in our culture system (**Figure 2**). The IC_50_ values of artesunate and artemether were 7.4 μM and 7.8 μM, respectively, indicating that both of the artemisinin derivatives showed higher inhibitory activity than artemisinin.

### Morphological Changes of Treated *B. duncani*

**Figure 3:**
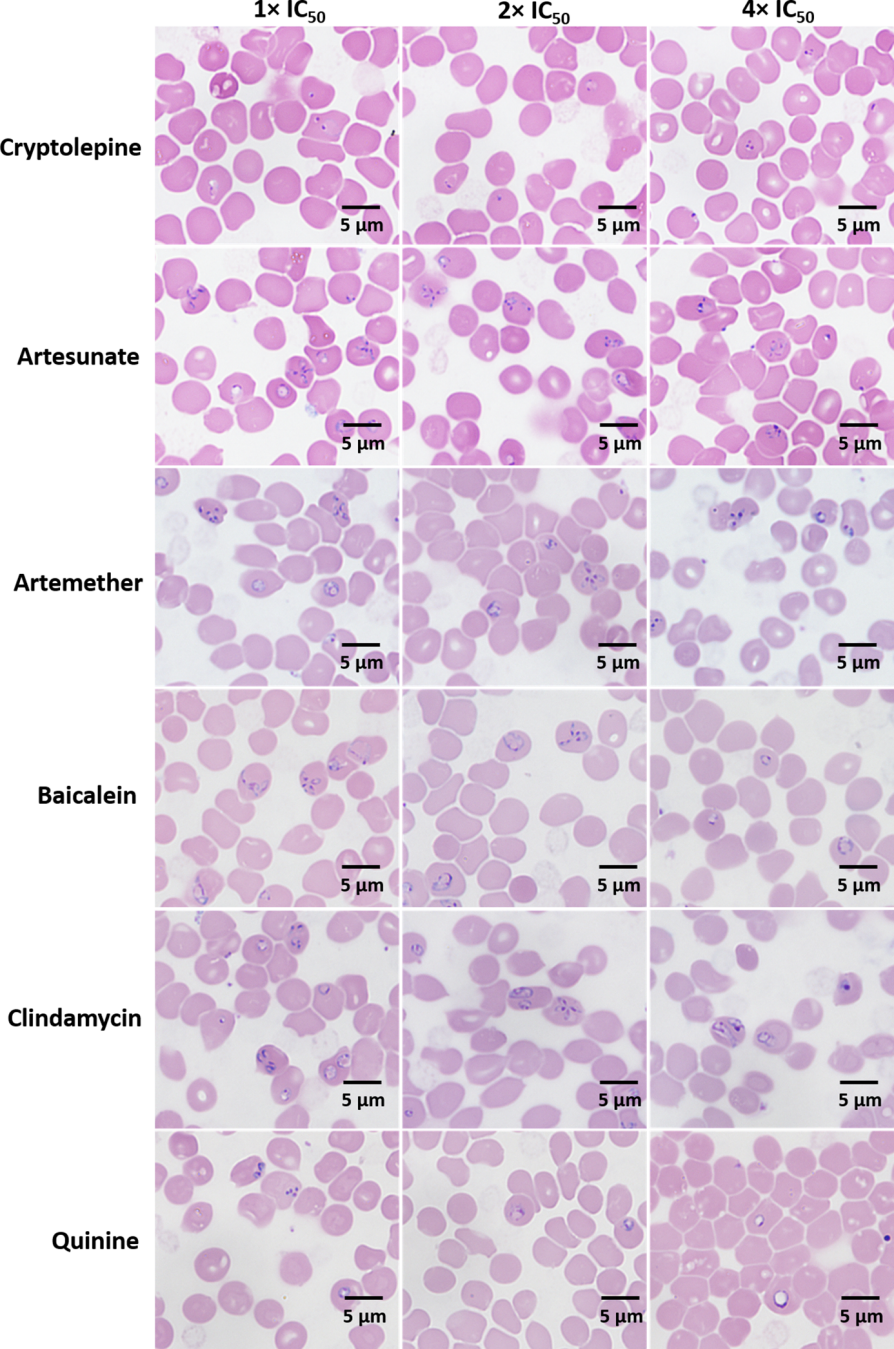
Morphology of *B. duncani* observed after treatment with cryptolepine, artesunate, artemether, baicalein, clindamycin, and quinine at their respective concentrations of 1×, 2×, and 4× IC_50_ values after three days of drug exposure.

After three days, the morphological changes of *B. duncani* treated with cryptolepine, artesunate, artemether, baicalein, clindamycin, and quinine at their respective concentrations of 1×, 2×, 4× IC_50_ values were observed. The parasites treated by cryptolepine (1×, 2×, 4× IC_50_ values) appeared to have dramatically condensed chromatin in both trophozoite and schizont stages (**Figure 3**). Parasites exposed to artesunate, artemether, and clindamycin at 4× IC_50_ values displayed teratogenic form, and those exposed to quinine showed gradually enlarged vacuole at the treated concentration of 1×, 2×, 4× IC_50_ values (**Figure 3**). *B. duncani* treated with artesunate (1×, 2× IC_50_ values), artemether (1×, 2×, IC_50_ values), baicalein (1×, 2×, 4× IC_50_ values), and clindamycin (1×, 2× IC_50_ values) did not show significant morphological changes (**Figure 3**).

### Subculture Studies to Evaluate Viability of *B. duncani* Treated With Herb Extract Compounds Artesunate, Artemether, Cryptolepine, Baicalein, as Well as Quinine, and Clindamycin

**Figure 4:**
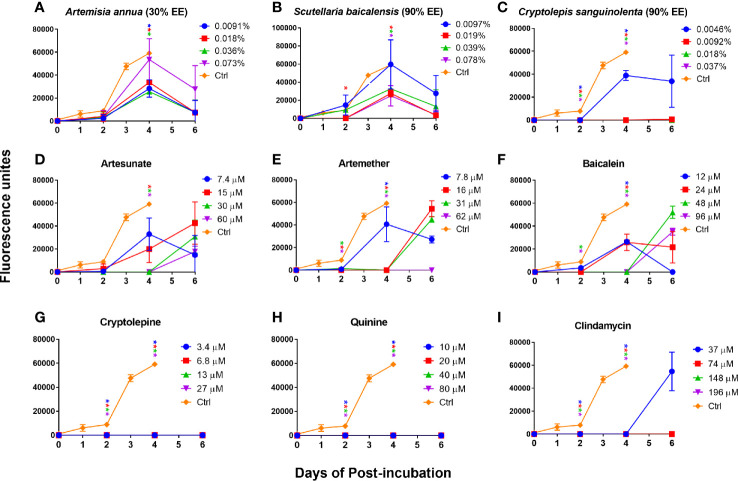
*In vitro* subculture of *B. duncani* in hamster erythrocytes after three days exposure with *Artemisia annua* **(A)**, *Scutellaria baicalensis* **(B)**, *Cryptolepis sanguinolenta* **(C)**, artesunate **(D)**, artemether **(E)**, baicalein **(F)**, cryptolepine **(G)**, quinine **(H)**, and clindamycin **(I)** at their respective concentrations of 1×, 2×, 4×, and 8× IC_50_ values. The cultures treated with 1% DMSO vehicle were set as control. The growth of the subculture was examined by SYBR Green I assay, and the fluorescence units at day zero were normalized as zero. Asterisks indicate the significant difference (*p* < 0.05) between the respective treated group and the untreated control. EE, ethanol extract.

In order to further confirm our finding that cryptolepine, baicalein, artesunate, and artemether were the most active compounds for inhibiting *B. duncani* growth *in vitro* and that their corresponding natural product extracts indeed have a killing effect, we performed subculture studies by re-inoculating three day treated infected erythrocytes into fresh uninfected erythrocytes to monitor regrowth. For comparison, we also evaluated the killing activity of quinine and clindamycin. The results showed that *Artemisia annua* (30% ethanol extract) and *Scutellaria baicalensis* (90% ethanol extract) treated *B. duncani* could regrow at 1×, 2×, 4×, and 8× IC_50_ values, and growth of most of these treated parasites could be observed at day two (**Figures 4A, B**). *Cryptolepis sanguinolenta* (90% ethanol extract) treated *B. duncani* regrew two days after treatment at 1× IC_50_ value, however, regrowth did not occur in six days after treatment at 2×, 4×, and 8× IC_50_ values (**Figure 4C**). Similarly, *B. duncani* treated with cryptolepine at 1×, 2×, 4×, and 8× IC_50_ values did not regrow in six days (**Figure 4G**). *B. duncani* treated with artesunate and artemether regrew after treatment at 1×, 2×, and 4× IC_50_ values, but no regrowth occurred after treatment with artemether at 8× IC_50_ (**Figures 4D, E**). Treatment with baicalein at an IC_50_ of 1× and 2× did not prevent *B. duncani* regrowth at day two of incubation, and regrowth also occurred at day four when treated with baicalein at an IC_50_ of 4× and 8× (**Figure 4F**). The drug quinine showed good killing effect (**Figure 4H**) at 1×, 2×, 4×, and 8× IC_50_ values, as did clindamycin at 2×, 4×, and 8× IC_50_ values. However, *B. duncani* could regrow after clindamycin treatment at 1× IC_50_ value (**Figure 4I**).

## Discussion

While recovery from clinical babesiosis can be spontaneous in healthy individuals, there is a much greater risk of severe symptoms and fatality in elderly, asplenic, or otherwise immunocompromised patients (Vannier and Krause, 2012; Vannier et al., 2015). Furthermore, some patients can experience persistent infection and clinical illness despite treatment with current therapeutic regimens (Krause et al., 1998; Raffalli and Wormser, 2016; Bloch et al., 2019). Importantly, illness can be more severe and recalcitrant to available treatment regimens when patients are simultaneously infected with multiple tickborne pathogens (Krause et al., 2002; Horowitz and Freeman, 2019; Sanchez-Vicente et al., 2019). In addition, some physicians opine that patients infected with *B. duncani* can have a more protracted course and typically require a longer duration of treatment than those infected with *B. microti* (Scott and Scott, 2018). The combination of quinine and clindamycin is the treatment regimen recommended for *B. duncani* spp. infections (Vannier and Krause, 2009) and all severe babesiosis infections (Centers for Disease, 1983). However, a clinical trial indicated that 72% of patients who received quinine plus clindamycin for babesiosis suffered side effects including tinnitus, vertigo, and gastrointestinal upset, in some cases severe enough to necessitate dosage decrease or treatment suspension (Krause et al., 2000). Previous studies found that quinine showed 30–100 nM of EC_50_ against Plasmodium falciparum (Skinner et al., 1996), and clindamycin showed around 8.8 nM of IC_50_ against Plasmodium falciparum 3D7 (Dahl and Rosenthal, 2007). However, in this study we found that quinine and clindamycin had much higher IC_50_ values against *B. duncani* growth in hamster erythrocytes (10 μM and 37 μM, respectively), and these results are comparable with research done *in vitro* in human erythrocytes (Abraham et al., 2018). Our subculture results showed quinine had good killing effect *in vitro* in the range of 10–80 μM (**Figure 4H**), but relapse occurred after the treatment of clindamycin at the concentration of 37 μM (**Figure 4I**). Importantly, we identified that the bioactive compounds derived from *Cryptolepis sanguinolenta*, *Artemisia annua*, and *Scutellaria baicalensis*, namely cryptolepine, artemisinin, artesunate, artemether, and baicalein (3.4 μM, 14 μM, 7.4 μM, 7.8 μM, and 12 μM, respectively) had comparable or even better activity against *B. duncani* than the commonly used medications quinine (10 μM) and clindamycin (37 μM).

Botanical medicine has a long documented history of use over 5,000 years (Borchardt, 2002) and recent retrospective and systematic reviews have concluded severe adverse events associated with botanical medicine usage are rare (Di Lorenzo et al., 2015; Restani et al., 2016). Research over the past few decades has demonstrated that many botanical medicines have antimicrobial, antiparasitic, and anticancer activity (Tagboto and Townson, 2001; Shoemaker et al., 2005; Feng et al., 2011). In our previous study, we identified seven herbal medicines that have good activity against stationary phase *Borrelia burgdorferi* compared to the control antibiotics used in clinical treatment (Feng J. et al., 2020). In humans, *Babesia* spp. frequently exist as co-infections with *Borrelia burgdorferi* through tick transmission (Magnarelli et al., 1995; Parveen and Bhanot, 2019). The screening results of the present study revealed that *Cryptolepis sanguinolenta*, *Artemisia annua*, and *Scutellaria baicalensis*, which previously showed good activity against *Borrelia burgdorferi* (Tona et al., 1998), also had impressive inhibitory effects against *B. duncani* (at even lower concentrations of 0.01%). Additionally, we showed that herbs extracted by different concentrations of alcohol (30%, 60%, and 90% ethanol extracts) had different inhibitory activity. Extracts of *Cryptolepis sanguinolenta* and *Scutellaria baicalensis* in higher concentrations of alcohol (90% and 60% ethanol extracts) had better inhibitory activity than in low alcohol concentration (30% ethanol extract), while *Artemisia annua* performed better at a lower alcohol concentration. This indicates that the corresponding bioactive ingredients of these herbs have diverse solubility in alcohol solvents, and that appropriate solvents and techniques are critical for the isolation and extraction of active constituents.

*Cryptolepis sanguinolenta* has been shown in preclinical studies to have anti-inflammatory, antimicrobial, anti-amoebic, anti-cancer, and anti-malarial properties (Grellier et al., 1996; Tona et al., 1998; Mills-Robertson et al., 2012; Ansah and Mensah, 2013; Hanprasertpong et al., 2014). Cryptolepine, an indoloquinoline alkaloid isolated from *Cryptolepis sanguinolenta*, has been shown *in vitro* to have significant antiplasmodial activity against drug-sensitive and drug-resistant strains (Osafo et al., 2017; Ameyaw et al., 2018), demonstrating an IC_50_ of 0.2–0.6 µM against *Plasmodium falciparum in vitro* (Grellier et al., 1996). *In vivo*, cryptolepine given orally at 50 mg/kg/day to mice infected with *Plasmodium berghei* was found to have moderate antimalarial activity to suppress parasitemia by 80% (Wright et al., 1996). The bioactivity of cryptolepine is believed to contribute to DNA intercalating activity targeting non-alternating CC sites, cell morphology change, and topoisomerase II inhibiting effects (Sawer et al., 1995; Bonjean et al., 1998; Lisgarten et al., 2002). Preliminary preclinical and clinical data indicate that *Cryptolepis sanguinolenta* and cryptolepine can have systemic effects *in vivo* and therefore are candidates for clinical development. Two open-label trials using different formulations of *Cryptolepis sanguinolenta* showed significant efficacy without observed toxicity in the treatment of patients with uncomplicated malaria (Bugyei et al., 2010; Tempesta, 2010). The pharmacokinetics of cryptolepine revealed that it has low bioavailability, a moderate half-life (4.5h) and extensive distribution (Forkuo et al., 2017a). A nanoformulation of cryptolepine exhibited a more favorable pharmacokinetic profile and a stronger antiplasmodial effect compared to the unaltered form of cryptolepine (Forkuo et al., 2017b). Toxicology data suggests that *Cryptolepis sanguinolenta* is generally safe at doses under 500 mg/kg of body weight (Osafo et al., 2017), however further studies are needed to evaluate concerns regarding potential anti-fertility and embryotoxic effects (Ajayi and Akhigbe, 2012; Mensah et al., 2019). Recently, Batiha et al. demonstrated that cryptolepine exhibited significant activity against multiple *Babesia* spp. *in vitro* and inhibited *B. microti* in mice at a dose of 5 mg/kg (Batiha et al., 2020b). To the best of our knowledge no study has reported the *in vitro* or *in vivo* treatment efficacy of whole herb *Cryptolepis sanguinolenta*, against *Babesia* spp. Among the panel of natural product extracts tested in this study, we found *Cryptolepis sanguinolenta* extract had the strongest inhibitory activity against *B. duncani in vitro* and no regrowth was visible after 6 days of subculture at doses of 2×, 4×, and 8× IC_50_ values. We further confirmed that cryptolepine is one important active constituent with a nearly three-fold lower IC_50_ value against *B. duncani* than quinine, and ten-fold lower than clindamycin. Importantly, the non-viability of *B. duncani* treated with cryptolepine at a concentration as low as 1× IC_50_ value (3.4 µM; **Figure 4G**) further suggests that cryptolepine may be a promising compound to treat human babesiosis. Further studies are needed to confirm this.

The Chinese herb, *Artemisia annua* (Sweet wormwood or Qing Hao) has been used for the treatment of malaria for centuries (Feng X. et al., 2020) and the Chinese scientist who isolated its most famous active constituent, artemisinin, was awarded the Nobel Prize in 2015 in recognition for artemisinin’s role in reducing malaria-associated morbidity and mortality (Liu and Liu, 2016). Artemisinin-based compounds have become pivotal in malaria treatment as they can reduce malarial parasitemia more rapidly than other known antimalarial drugs and are effective against all stages of *Plasmodium* spp (Meshnick et al., 1996; Olliaro et al., 2001). The mechanisms of action of artemisinin compounds are incompletely understood (Krause et al., 2007), although it has been proposed to be related to their ability to generate free radicals which can damage pathogen DNA and proteins (Eckstein-Ludwig et al., 2003; Wang et al., 2015). The safety, tolerability, and systemic effects of artemisinin-based compounds have been well-documented over decades of malarial clinical trials (Price et al., 1999; Adjuik et al., 2004; World Health Organization (WHO), 2015) including treatment during the 2^nd^ and 3^rd^ trimesters of pregnancy (Kovacs et al., 2016) and possibly during the first trimester (D’Alessandro et al., 2020). The safety, tolerability, and systemic effects of the whole herb, *Artemisia annua*, have also been documented, although in more limited clinical studies (Hunt et al., 2016, Stebbings et al., 2016; Yang et al., 2017). *Babesia* spp. and *Plasmodium* spp. share a similar intraerythrocytic life cycle and pathobiology which partly explains why artemisinin and its derivatives were able to inhibit *in vitro* or *in vivo* growth of *B. gibsoni*, *B. equi*, *B. bigemina*, *B. bovis*, and *B. microti* (Kumar et al., 2003; Goo et al., 2010; Mazuz et al., 2013; Iguchi et al., 2015). In our study, we found both 30% and 60% ethanol extracts of *Artemisia annua* had good inhibitory activity against *B. duncani in vitro*. The IC_50_ values of 30%, 60%, and 90% ethanol extracts of *Artemisia annua* were 0.0091%, 0.0097%, and 0.030%, respectively. A previous study indicated that flavonoids from *Artemisia annua* have potential synergistic anti-malaria effects with artemisinin (Ferreira et al., 2010). Because the content of artemisinin in the *Artemisia annua* samples used for extraction in this study is low (0.11% as tested by high-performance liquid chromatography/UV-visual spectroscopy at the Institute for Food Safety and Defense, Centralia, WA), the role of anti-*Babesia* effects of other bioactive constituents such as flavonoids in *Artemisia annua* should not be ignored. Indeed, *Artemisia* spp. have documented antiparasitic effects beyond their artemisinin content. *Artemisia afra*, a non-artemisinin-containing species, was recently found to have similar efficacy as *Artemisia annua*, an artemisinin-containing species, in the treatment of malaria and schistosomiasis (Munyangi et al., 2018; Munyangi et al., 2019). Our study indicated that artemisinin and artemisinin derivatives (artesunate and artemether) alone also had *in vitro* inhibitory effects against *B. duncani.* Specifically, we found the IC_50_ values of artesunate and artemether were lower than that of quinine and clindamycin. It has been reported that *B. gibsoni* (wild type) was more susceptible to artemisinin than artesunate and artemether. Interestingly, the present study showed artesunate and artemether were more effective than artemisinin against *B. duncani* (**Figure 2**), and the IC_50_ values of artemisinin, artesunate, and artemether in this study were much higher than their corresponding IC_50_ values (nanomolar) *in vitro* against *B. gibsoni* (Matsuu et al., 2008; Iguchi et al., 2015). A previous study showed that 1 µg/ml (0.5 µM, approximately) of artesunate was sufficient to eradicate *B. equi in vitro* which was not able to regrow four days after drug withdrawal (Nagai et al., 2003). In contrast, our subculture study showed the regrowth of *B. duncani* could occur six days after treatment of artesunate at as high as 60 µM (8× IC_50_), and of artesunate at 31 µM (4× IC_50_). Because there is limited data in the research literature on the efficacy of artemisinin-related drugs against other *Babesia* spp., it remains unknown whether the low susceptibility to artemisinin, artesunate, and artemether is specific to *B. duncani*. Preliminary animal data suggest that adding a second anti-parasitic agent, such as cryptolepine from *Cryptolepis sanguinolenta*, may increase the susceptibility of artemisinin-based therapy to *Babesia* spp., similar to the use of two or three agents in artemisinin-based combination therapy (ACT) for malaria (Kumar et al., 2003; Iguchi et al., 2015; Forkuo et al., 2016; Carvalho et al., 2020). The development of malarial resistance to ACT raises important points regarding the potential benefits of using a whole plant form (e.g., *Artemisia annua)* vs a single constituent form (e.g., artemisinin) (Haldar et al., 2018). Although theoretic concerns that *Artemisia annua’s* low artemisinin content may increase the risk of antimicrobial resistance (W.H.O.W.P.S, 2012) subsequent testing has revealed that antimicrobial resistance occurred three times slower when a whole plant form of *Artemisia annua* was used compared to artemisinin monotherapy (Elfawal et al., 2015). Additionally, preliminary clinical studies have revealed that treatment with whole plant *Artemisia annua* was associated with improved clinical outcomes and fewer side effects compared to ACT (Daddy et al., 2017; Munyangi et al., 2019). The improved outcomes when using a whole plant form of *Artemisia annua* can be explained by an evolutionarily guided synergism among its phytochemicals resulting in increased bioavailability of active compounds (pharmacodynamic synergy), complementary mechanisms of action (antimicrobial, immune-modulating, anti-inflammatory, etc.), multidrug resistance inhibition, and multiple antimicrobial compounds (Rasoanaivo et al., 2011; Munyangi et al., 2019; Caesar and Cech, 2019; Desrosiers et al., 2020, Zhao et al., 2020).

*Scutellaria baicalensis* (Chinese skullcap) has been widely used as a medicinal plant in China for thousands of years for the treatment of diarrhea, dysentery, hypertension, hemorrhage, insomnia, inflammation and respiratory infections (Zhao et al., 2016). Over forty different compounds have been isolated from *Scutellaria baicalensis* and the antimicrobial activity is hypothesized to be due to flavonoids, volatile oils, terpenoids, and polysaccharides (Wu et al., 2008; Lu et al., 2011; Leung et al., 2016). Previous studies have demonstrated that *Scutellaria baicalensis* extract and one of its primary bioactive constituents, baicalein, exhibited *in vitro* activity against various morphologic forms of *Borrelia burgdorferi* and *Borrelia garinii* (Goc et al., 2015; Feng J. et al., 2020). In traditional Korean medicine, *Scutellaria baicalensis* was combined with *Artemisia apiacea* for the treatment of malaria and other malaria-like diseases (Trinh et al., 2018). Existing clinical trial data on *Scutellaria baicalensis* indicates it is non-toxic and has systemic effects showing benefits in diabetes, enterovirus encephalitis, and adjunctive cancer care (Smol’ianinov et al., 1997; Lin et al., 2016; Shin et al., 2020). A medical food combination of concentrated baicalin and catechin (Limbrel™, Move Free Advanced™) was linked to reversible liver damage in at least 35 cases (Chalasani et al., 2012), however a recent study found no hepatotoxicity in patients taking a whole herb formulation of *Scutellaria baicalensis* dosed at 1,335 mg per day for an average of 444 days (Puri et al., 2019). Human safety studies on baicalin have shown that doses between 200–800 mg per day are safe and well-tolerated with a favorable pharmacokinetic profile (Li et al., 2014; Pang et al., 2016). The anti-babesial activity of *Scutellaria baicalensis* and its active constituents have not been previously reported. In this study, we found *Scutellaria baicalensis* and baicalein showed good *in vitro* activity against *B. duncani*, with the IC_50_ value of baicalein practically the same as quinine (12 µM vs 10 µM), and three times lower than clindamycin (12 µM vs 37 µM). A study of ethanol extracts from *Scutellaria baicalensis* showed that it had the strongest inhibitory and bactericidal efficacy against six foodborne pathogens and had the highest content of flavonoids and phenolic acids (Lu et al., 2011). These findings are consistent with the present study which demonstrated *Scutellaria baicalensis* extract in 90% ethanol had a lower IC_50_ against *B. duncani* than those in 30% and 60% ethanol. Similar to artesunate, regrowth of *B. duncani* treated with baicalein did not occur in four days at 4× and 8× IC_50_ values.

It should be noted that *Alchornea cordifolia* and *Polygonum cuspidatum* extracts also showed good inhibitory effect against *B. duncani* in our study. Both have been documented to have antimicrobial and anti-inflammatory activity (Ebi, 2001; Manga et al., 2004; Shan et al., 2008; Ghanim et al., 2010). *Alchornea cordifolia* has been used by traditional herbalists in several African countries for the treatment of malaria (Boniface et al., 2016) and pre-clinical studies corroborate significant anti-malarial effects (Mustofa et al., 2000; Mesia et al., 2008; Ayisi et al., 2011). The active constituents of *Alchornea cordifolia* extract are complex, including phenolic acid, gallic acid, protocatechuic acid, ellagic acid, and quercetin (Boniface et al., 2016). Of note, ellagic acid has previously shown *in vitro* antiplasmodial activity (Lamikanra et al., 1990; Banzouzi et al., 2002) and *in vivo* antibabesial activity (Beshbishy et al., 2019) and quercetin has been shown to have antiplasmodial and antibabesial effects (Batiha et al., 2020a). Although clinical data is lacking, preclinical studies reveal *Alchornea cordifolia* has favorable toxicology and bioavailability profiles (Gatsing et al., 2010; Ajibade and Olayemi, 2015; Boniface et al., 2016; Djimeli et al., 2017). *Polygonum cuspidatum* contains over 60 active constituents with wide-ranging mechanisms of action and varying degrees of bioavailability (Pan et al., 2019). Stilbenes (e.g., resveratrol) and hydroxyanthraquinones (e.g., emodin) are among the active constituents of *Polygonum cuspidatum* that exert antibacterial properties (Shan et al., 2008). The bioavailability of orally administered resveratrol and emodin is suboptimal (Walle, 2011; Dong et al., 2016) and therefore numerous strategies have been developed to improve their pharmacokinetic profiles (Chimento et al., 2019; Dewanjee et al., 2020). Despite its low bioavailability, resveratrol has high bioactivity which is thought to be related to extensive biotransformation in enterocytes, hepatocytes, and the gastrointestinal microbiome (Luca et al., 2020). Although various active constituents from *Polygonum cuspidatum* have been shown to have antiparasitic effects (Passos et al., 2015; Lin et al., 2017), to our knowledge, this is the first time that the antibabesial effects of *Polygonum cuspidatum* have been reported. Interestingly, our previous study indicated that *Cryptolepis sanguinolenta*, *Artemisia annua*, *Scutellaria baicalensis*, and *Polygonum cuspidatum* extracts have activity against *Borrelia burgdorferi* (Feng J. et al., 2020), indicating that it may be advantageous to use these herbs to simultaneously target these different pathogens in complex Lyme disease with co-infections.

A potential limitation of the study is the descriptive nature of the study. Future studies are needed to determine the active components of the effective hits, to better investigate their effects on *Babesia* morphology through microscopy techniques, and to characterize if extracts produce *Babesia* death or just inhibit its growth or cell cycle, and to confirm their activity in animal models.

## Conclusions

In this study we identified herbal medicines *Cryptolepis sanguinolenta*, *Artemisia annua*, *Scutellaria baicalensis*, *Alchornea cordifolia*, and *Polygonum cuspidatum* that exhibit good *in vitro* inhibitory activity against *B. duncani* at 0.01% (v/v). Furthermore, we tested the activity of the potential bioactive constituents and found that cryptolepine, artemisinin-related compounds, and baicalein may be promising alternatives to treat human babesiosis caused by *B. duncani*. These botanical medicines can be considered candidates for clinical development based on their low cost, favorable toxicity and pharmacokinetic profiles and history of safety and efficacy from clinical trials and/or use in traditional medicine. Future research will be needed to identify the bioactive components and to test if they also have activity against other *Babesia* species *in vitro* and *in vivo*. In order to identify more effective treatments, combinations of these active herbs and active compounds need to be tested both *in vitro* and in animal models. This research needs to be done both in isolation and potentially in combination with current antibabesial drugs. Since the botanical medicines *Cryptolepis sanguinolenta*, *Artemisia annua*, *Scutellaria baicalensis, Alchornea cordifolia*, and *Polygonum cuspidatum* are already in clinical use, it is also important for future studies to evaluate them directly in patients using specific clinical treatment regimens.

## Data Availability Statement

The original contributions presented in the study are included in the article/**Supplementary Material**. Further inquiries can be directed to the corresponding author.

## Ethics Statement

The animal study was reviewed and approved by Johns Hopkins Institutional Animal Care and Use Committee.

## Author Contributions

Conceptualization, YMZ and YZ. Methodology, YMZ, YZ, HA-M, and JL. Data curation, YMZ and HA-M. Funding acquisition, YZ. Writing—original draft, YMZ. Writing—review and editing, JL, SS, and YZ. All authors contributed to the article and approved the submitted version.

## Funding

Sources of funding included Bay Area Lyme Foundation, the Steven and Alexandra Cohen Foundation, Global Lyme Alliance, NatCapLyme, LivLyme Foundation, and the Einstein–Sim Family Charitable Fund.

## Conflict of Interest

JL is the owner of two naturopathic medical practices, FOCUS Health Group and Door One Concierge, which provides treatment to patients with tick-borne diseases. JL does receive profits from medical services and botanical preparations he exclusively makes available to patients in these two practices and does not currently sell botanical products commercially.

The remaining authors declare that the research was conducted in the absence of any commercial or financial relationships that could be construed as a potential conflict of interest.

## References

[B1] Abraham A. Brasov I. Thekkiniath J. Kilian N. Lawres L. Gao R.. (2018). Establishment of a continuous in vitro culture of *Babesia duncani* in human erythrocytes reveals unusually high tolerance to recommended therapies. J. Biol. Chem. 293, 19974–19981. 10.1074/jbc.AC118.005771 30463941 PMC6311517

[B2] Adjuik M. Babiker A. Garner P. Olliaro P. Taylor W. White N.. (2004). Artesunate combinations for treatment of malaria: meta-analysis. Lancet 363, 9–17. 10.1016/S0140-6736(03)15162-8 14723987

[B3] Ajayi A. F. Akhigbe R. E. (2012). Antifertility activity of *Cryptolepis sanguinolenta* leaf ethanolic extract in male rats. J. Hum. Reprod. Sci. 5, 43–47. 10.4103/0974-1208.97799 22870014 PMC3409919

[B4] Ajibade T. O. Olayemi F. O. (2015). Reproductive and toxic effects of methanol extract of *Alchornea cordifolia* leaf in male rats. Andrologia 47, 1034–1040. 10.1111/and.12374 25418355

[B5] Ameyaw E. O. Asmah K. B. Biney R. P. Henneh I. T. Owusu-Agyei P. Prah J.. (2018). Isobolographic analysis of co-administration of two plant-derived antiplasmodial drug candidates, cryptolepine and xylopic acid, in *Plasmodium berghei* . Malar. J. 17, 153. 10.1186/s12936-018-2283-8 29618354 PMC5885295

[B6] Ansah C. Mensah K. B. (2013). A review of the anticancer potential of the antimalarial herbal *Cryptolepis sanguinolenta* and its major alkaloid cryptolepine. Ghana Med. J. 47, 137–147. 24391229 PMC3875281

[B7] Ayisi N. K. Appiah-Opong R. Gyan B. Bugyei K. Ekuban F. (2011). *Plasmodium falciparum*: Assessment of Selectivity of Action of Chloroquine, Alchornea cordifolia, Ficus polita, and Other Drugs by a Tetrazolium-Based Colorimetric Assay. Malar. Res. Treat 2011, 816250. 10.4061/2011/816250 22312574 PMC3265290

[B8] Banzouzi J. T. Prado R. Menan H. Valentin A. Roumestan C. Mallie M.. (2002). In vitro antiplasmodial activity of extracts of *Alchornea cordifolia* and identification of an active constituent: ellagic acid. J. Ethnopharmacol. 81, 399–401. 10.1016/S0378-8741(02)00121-6 12127243

[B9] Batiha G. E. Beshbishy A. M. Ikram M. Mulla Z. S. El-Hack M. E. A. Taha A. E.. (2020a). The Pharmacological Activity, Biochemical Properties, and Pharmacokinetics of the Major Natural Polyphenolic Flavonoid: Quercetin. Foods 9. 10.3390/foods9030374 PMC7143931 32210182

[B10] Batiha G. E. Beshbishy A. M. Alkazmi L. M. Nadwa E. H. Rashwan E. K. Yokoyama N.. (2020b). In vitro and in vivo growth inhibitory activities of cryptolepine hydrate against several Babesia species and Theileria equi. PLoS. Negl. Trop. Dis. 14 (8), e0008489. 10.1371/journal.pntd.0008489 PMC7451656 32853247

[B11] Beshbishy A. M. Batiha G. E. Yokoyama N. Igarashi I. (2019). Ellagic acid microspheres restrict the growth of Babesia and Theileria in vitro and *Babesia microti in* vivo. Parasit. Vectors 12, 269. 10.1186/s13071-019-3520-x 31138282 PMC6537213

[B12] Birkenheuer A. J. Whittington J. Neel J. Large E. Barger A. Levy M. G.. (2006). Molecular characterization of a Babesia species identified in a North American raccoon. J. Wildl. Dis. 42, 375–380. 10.7589/0090-3558-42.2.375 16870860

[B13] Bloch E. M. Kumar S. Krause P. J. (2019). Persistence of *Babesia microti* Infection in Humans. Pathogens 8 (3), 102. 10.3390/pathogens8030102 PMC6789900 31319461

[B14] Boniface P. K. Ferreira S. B. Kaiser C. R. (2016). Recent trends in phytochemistry, ethnobotany and pharmacological significance of *Alchornea cordifolia* (Schumach. & Thonn.) Muell. Arg. J. Ethnopharmacol. 191, 216–244. 10.1016/j.jep.2016.06.021 27296085

[B15] Bonjean K. De Pauw-Gillet M. C. Defresne M. P. Colson P. Houssier C. Dassonneville L.. (1998). The DNA intercalating alkaloid cryptolepine interferes with topoisomerase II and inhibits primarily DNA synthesis in B16 melanoma cells. Biochemistry 37, 5136–5146. 10.1021/bi972927q 9548744

[B16] Borchardt J. K. (2002). The Beginnings of Drug Therapy: Ancient Mesopotamian Medicine. Drug News Perspect. 15, 187–192. 10.1358/dnp.2002.15.3.840015 12677263

[B17] Bugyei K. A. Boye G. L. Addy M. E. (2010). Clinical efficacy of a tea-bag formulation of *Cryptolepis sanguinolenta* root in the treatment of acute uncomplicated falciparum malaria. Ghana Med. J. 44, 3–9. 10.4314/gmj.v44i1.68849 21326984 PMC2956309

[B18] Buhner S. H. (2015). Natural Treatments For Lyme Coinfections: Anaplasma, Babesia, And Ehrlichia (Rochester, Vermont: Healing Arts Press).

[B19] Caesar L. K. Cech N. B. (2019). Synergy and antagonism in natural product extracts: when 1 + 1 does not equal 2. Nat. Prod. Rep. 36, 869–888. 10.1039/C9NP00011A 31187844 PMC6820002

[B20] Carvalho L. J. M. Tuvshintulga B. Nugraha A. B. Sivakumar T. Yokoyama N. (2020). Activities of artesunate-based combinations and tafenoquine against *Babesia bovis* in vitro and *Babesia microti* in vivo. Parasit. Vectors 13, 362. 10.1186/s13071-020-04235-7 32690081 PMC7372749

[B21] Centers for Disease Control (CDC). (1983). Clindamycin and quinine treatment for *Babesia microti* infections. MMWR Morb. Mortal. Wkly. Rep. 32, 65–6, 72. 6405180

[B22] Chalasani N. Vuppalanchi R. Navarro V. Fontana R. Bonkovsky H. Barnhart H.. (2012). Acute Liver Injury due to Flavocoxid (Limbrel), a Medical Food for Osteoarthritis. Ann. Internal Med. 156, 857–U129. 10.7326/0003-4819-156-12-201206190-00006 22711078 PMC3825458

[B23] Chimento A. De Amicis F. Sirianni R. Sinicropi M. S. Puoci F. Casaburi I.. (2019). Progress to Improve Oral Bioavailability and Beneficial Effects of Resveratrol. Int. J. Mol. Sci. 20 (6), 1381. 10.3390/ijms20061381 PMC6471659 30893846

[B24] Cimanga K. De Bruyne T. Lasure A. Van Poel B. Pieters L. Claeys M.. (1996). In vitro biological activities of alkaloids from *Cryptolepis sanguinolenta* . Planta Med. 62, 22–27. 10.1055/s-2006-957789 8720383

[B25] Daddy N. B. Kalisya L. M. Bagire P. G. Watt R. L. Towler M. J. Weathers P. J. (2017). *Artemisia annua* dried leaf tablets treated malaria resistant to ACT and i.v. artesunate: Case reports. Phytomedicine 32, 37–40. 10.1016/j.phymed.2017.04.006 28732806 PMC5547396

[B26] Dahl E. L. Rosenthal P. J. (2007). Multiple antibiotics exert delayed effects against the *Plasmodium falciparum* apicoplast. Antimicrob. Agents Chemother. 51, 3485–3490. 10.1128/AAC.00527-07 17698630 PMC2043295

[B27] Desrosiers M. R. Mittelman A. Weathers P. J. (2020). Dried Leaf *Artemisia Annua* Improves Bioavailability of Artemisinin via Cytochrome P450 Inhibition and Enhances Artemisinin Efficacy Downstream. Biomolecules 10 (2), 254. 10.3390/biom10020254 PMC7072484 32046156

[B28] Dewanjee S. Chakraborty P. Mukherjee B. De Feo V. (2020). Plant-Based Antidiabetic Nanoformulations: The Emerging Paradigm for Effective Therapy. Int. J. Mol. Sci. 21 (6), 2217. 10.3390/ijms21062217 PMC7139625 32210082

[B29] Di Lorenzo C. Ceschi A. Kupferschmidt H. Lude S. De Souza Nascimento E. Dos Santos A.. (2015). Adverse effects of plant food supplements and botanical preparations: a systematic review with critical evaluation of causality. Br. J. Clin. Pharmacol. 79, 578–592. 10.1111/bcp.12519 25251944 PMC4386943

[B30] Diuk-Wasser M. A. Vannier E. Krause P. J. (2016). Coinfection by Ixodes Tick-Borne Pathogens: Ecological, Epidemiological, and Clinical Consequences. Trends Parasitol. 32, 30–42. 10.1016/j.pt.2015.09.008 26613664 PMC4713283

[B31] Djimeli M. N. Fodouop S. P. C. Njateng G. S. S. Fokunang C. Tala D. S. Kengni F.. (2017). Antibacterial activities and toxicological study of the aqueous extract from leaves of *Alchornea cordifolia* (Euphorbiaceae). BMC Complement. Altern. Med. 17, 349. 10.1186/s12906-017-1854-5 28676114 PMC5496605

[B32] Dong X. Fu J. Yin X. Cao S. Li X. Lin L.. (2016). Emodin: A Review of its Pharmacology, Toxicity and Pharmacokinetics. Phytother. Res. 30, 1207–1218. 10.1002/ptr.5631 27188216 PMC7168079

[B33] D’Alessandro S. Menegola E. Parapini S. Taramelli D. Basilico N. (2020). Safety of Artemisinin Derivatives in the First Trimester of Pregnancy: A Controversial Story. Molecules 25 (15), 3505. 10.3390/molecules25153505 PMC7435965 32752056

[B34] Ebi G. C. (2001). Antimicrobial activities of *Alchornea cordifolia* . Fitoterapia 72, 69–72. 10.1016/S0367-326X(00)00254-9 11163946

[B35] Eckstein-Ludwig U. Webb R. J. Van Goethem I. D. East J. M. Lee A. G. Kimura M.. (2003). Artemisinins target the SERCA of *Plasmodium falciparum* . Nature 424, 957–961. 10.1038/nature01813 12931192

[B36] Elfawal M. A. Towler M. J. Reich N. G. Weathers P. J. Rich S. M. (2015). Dried whole-plant Artemisia annua slows evolution of malaria drug resistance and overcomes resistance to artemisinin. Proc. Natl. Acad. Sci. U.S.A. 112, 821–826. 10.1073/pnas.1413127112 25561559 PMC4311864

[B37] Feng Y. Wang N. Zhu M. Feng Y. Li H. Tsao S. (2011). Recent progress on anticancer candidates in patents of herbal medicinal products. Recent Pat. Food Nutr. Agric. 3, 30–48. 10.2174/2212798411103010030 21114469

[B38] Feng J. Leone J. Schweig S. Zhang Y. (2020). Evaluation of Natural and Botanical Medicines for Activity Against Growing and Non-growing Forms of *B* . Burgdorferi. Front. Med. (Lausanne) 7, 6. 10.3389/fmed.2020.00006 32154254 PMC7050641

[B39] Feng X. Cao S. Qiu F. Zhang B. (2020). Traditional application and modern pharmacological research of *Artemisia annua* L. Pharmacol. Ther., 16, 107650. 10.1016/j.pharmthera.2020.107650 32758647

[B40] Ferreira J. F. S. Luthria D. L. Sasaki T. Heyerick A. (2010). Flavonoids from *Artemisia annua* L. as Antioxidants and Their Potential Synergism with Artemisinin against Malaria and Cancer. Molecules 15, 3135–3170. 10.3390/molecules15053135 20657468 PMC6263261

[B41] Forkuo A. D. Ansah C. Boadu K. M. Boampong J. N. Ameyaw E. O. Gyan B. A.. (2016). Synergistic anti-malarial action of cryptolepine and artemisinins. Malar. J. 15, 89. 10.1186/s12936-016-1137-5 26879905 PMC4754817

[B42] Forkuo A. D. Ansah C. Pearson D. Gertsch W. Cirello A. Amaral A.. (2017a). Identification of cryptolepine metabolites in rat and human hepatocytes and metabolism and pharmacokinetics of cryptolepine in Sprague Dawley rats. BMC Pharmacol. Toxicol. 18, 84. 10.1186/s40360-017-0188-8 29273084 PMC5741962

[B43] Forkuo A. D. Ansah C. Mensah K. B. Annan K. Gyan B. Theron A.. (2017b). In vitro anti-malarial interaction and gametocytocidal activity of cryptolepine. Malar. J. 16, 496. 10.1186/s12936-017-2142-z 29282057 PMC5745596

[B44] Gatsing D. Nkeugouapi C. F. N. Nji-Nkah B. F. Kuiate J. R. Tchouanguep F. M. (2010). Antibacterial Activity, Bioavailability and Acute Toxicity Evaluation of the Leaf Extract of *Alchornea cordifolia* (Euphorbiaceae). Int. J. Pharmacol. 6, 173–182. 10.3923/ijp.2010.173.182

[B45] Ghanim H. Sia C. L. Abuaysheh S. Korzeniewski K. Patnaik P. Marumganti A.. (2010). An antiinflammatory and reactive oxygen species suppressive effects of an extract of *Polygonum cuspidatum* containing resveratrol. J. Clin. Endocrinol. Metab. 95, E1–E8. 10.1210/mend.24.7.9998 20534755 PMC2936054

[B46] Goc A. Niedzwiecki A. Rath M. (2015). In vitro evaluation of antibacterial activity of phytochemicals and micronutrients against *Borrelia burgdorferi* and *Borrelia garinii* . J. Appl. Microbiol. 119, 1561–1572. 10.1111/jam.12970 26457476 PMC4738477

[B47] Gonzalez L. M. Rojo S. Gonzalez-Camacho F. Luque D. Lobo C. A. Montero E. (2014). Severe babesiosis in immunocompetent man, Spain, 2011. Emerg. Infect. Dis. 20, 724–726. 10.3201/eid2004.131409 24656155 PMC3966382

[B48] Goo Y. K. Terkawi M. A. Jia H. Aboge G. O. Ooka H. Nelson B.. (2010). Artesunate, a potential drug for treatment of Babesia infection. Parasitol. Int. 59, 481–486. 10.1016/j.parint.2010.06.004 20541037

[B49] Grellier P. Ramiaramanana L. Millerioux V. Deharo E. Schrevel J. Frappier F.. (1996). Antimalarial activity of cryptolepine and isocryptolepine, alkaloids isolated from *Cryptolepis sanguinolenta* . Phytother. Res. 10, 317–321. 10.1002/(SICI)1099-1573(199606)10:4<317::AID-PTR858>3.0.CO;2-0

[B50] Haldar K. Bhattacharjee S. Safeukui I. (2018). Drug resistance in Plasmodium. Nat. Rev. Microbiol. 16, 156–170. 10.1038/nrmicro.2017.161 29355852 PMC6371404

[B51] Hanprasertpong N. Teekachunhatean S. Chaiwongsa R. Ongchai S. Kunanusorn P. Sangdee C.. (2014). Analgesic, anti-inflammatory, and chondroprotective activities of *Cryptolepis buchanani* extract: in vitro and in vivo studies. BioMed. Res. Int. 2014, 978582. 10.1155/2014/978582 25247198 PMC4160634

[B52] Horowitz R. I. Freeman P. R. (2019). Precision medicine: retrospective chart review and data analysis of 200 patients on dapsone combination therapy for chronic Lyme disease/post-treatment Lyme disease syndrome: part 1. Int. J. Gen. Med. 12, 101–119. 10.2147/IJGM.S193608 30863136 PMC6388746

[B53] Hunt S. Stebbings S. McNamara D. (2016). An open-label six-month extension study to investigate the safety and efficacy of an extract of *Artemisia annua* for managing pain, stiffness and functional limitation associated with osteoarthritis of the hip and knee. N. Z. Med. J. 129, 97–102. 27806033

[B54] Iguchi A. Matsuu A. Matsuyama K. Hikasa Y. (2015). The efficacy of artemisinin, artemether, and lumefantrine against *Babesia gibsoni* in vitro. Parasitol. Int. 64, 190–193. 10.1016/j.parint.2014.12.006 25523292

[B55] Jaiswal Y. Liang Z. Zhao Z. (2016). Botanical drugs in Ayurveda and Traditional Chinese Medicine. J. Ethnopharmacol. 194, 245–259. 10.1016/j.jep.2016.06.052 27394388

[B56] Kjemtrup A. M. Conrad P. A. (2000). Human babesiosis: an emerging tick-borne disease. Int. J. Parasitol. 30, 1323–1337. 10.1016/S0020-7519(00)00137-5 11113258

[B57] Klayman D. L. (1993). *Artemisia annua* - from Weed To Respectable Antimalarial Plant. ACS Sym. Ser. 534, 242–255. 10.1021/bk-1993-0534.ch017

[B58] Kovacs S. D. van Eijk A. M. Sevene E. Dellicour S. Weiss N. S. Emerson S.. (2016). The Safety of Artemisinin Derivatives for the Treatment of Malaria in the 2nd or 3rd Trimester of Pregnancy: A Systematic Review and Meta-Analysis. PloS One 11, e0164963. 10.1371/journal.pone.0164963 27824884 PMC5100961

[B59] Krause P. J. Telford S. R. 3. Spielman A. Sikand V. Ryan R. Christianson D.. (1996). Concurrent Lyme disease and babesiosis. Evidence for increased severity and duration of illness. JAMA 275, 1657–1660. 10.1001/jama.1996.03530450047031 8637139

[B60] Krause P. J. Spielman A. Telford S. R. 3. Sikand V. K. McKay K. Christianson D.. (1998). Persistent parasitemia after acute babesiosis. N. Engl. J. Med. 339, 160–165. 10.1056/NEJM199807163390304 9664092

[B61] Krause P. J. Lepore T. Sikand V. K. Gadbaw J. Jr. Burke G. Telford S. R. 3.. (2000). Atovaquone and azithromycin for the treatment of babesiosis. N. Engl. J. Med. 343, 1454–1458. 10.1056/NEJM200011163432004 11078770

[B62] Krause P. J. McKay K. Thompson C. A. Sikand V. K. Lentz R. Lepore T.. (2002). Disease-specific diagnosis of coinfecting tickborne zoonoses: babesiosis, human granulocytic ehrlichiosis, and Lyme disease. Clin. Infect. Dis. 34, 1184–1191. 10.1086/339813 11941544

[B63] Krause P. J. Daily J. Telford S. R. Vannier E. Lantos P. Spielman A. (2007). Shared features in the pathobiology of babesiosis and malaria. Trends Parasitol. 23, 605–610. 10.1016/j.pt.2007.09.005 17988944

[B64] Krause P. J. Gewurz B. E. Hill D. Marty F. M. Vannier E. Foppa I. M.. (2008). Persistent and relapsing babesiosis in immunocompromised patients. Clin. Infect. Dis. 46, 370–376. 10.1086/525852 18181735

[B65] Kumar S. Gupta A. K. Pal Y. Dwivedi S. K. (2003). In-vivo therapeutic efficacy trial with artemisinin derivative, buparvaquone and imidocarb dipropionate against *Babesia equi* infection in donkeys. J. Vet. Med. Sci. 65, 1171–1177. 10.1292/jvms.65.1171 14665744

[B66] Lamikanra A. Ogundaini A. O. Ogungbamila F. O. (1990). Antibacterial Constituents of Alchornea-Cordifolia Leaves. Phytother. Res. 4, 198–200. 10.1002/ptr.2650040508 10189955

[B67] Leiby D. A. (2006). Babesiosis and blood transfusion: flying under the radar. Vox. Sang. 90, 157–165. 10.1111/j.1423-0410.2006.00740.x 16507014 PMC7169304

[B68] Leung K. C. Seneviratne C. J. Li X. Leung P. C. Lau C. B. Wong C. H.. (2016). Synergistic Antibacterial Effects of Nanoparticles Encapsulated with *Scutellaria baicalensis* and Pure Chlorhexidine on Oral Bacterial Biofilms. Nanomater. (Basel) 6 (4), 61. 10.3390/nano6040061 PMC5302556 28335189

[B69] Li M. Shi A. Pang H. Xue W. Li Y. Cao G.. (2014). Safety, tolerability, and pharmacokinetics of a single ascending dose of baicalein chewable tablets in healthy subjects. J. Ethnopharmacol. 156, 210–215. 10.1016/j.jep.2014.08.031 25219601

[B70] Li Y. Liu M. Rizk M. A. Moumouni P. F. A. Lee S. H. Galon E. M.. (2020). Drug screening of food and drug administration-approved compounds against *Babesia bovis* in vitro. Exp. Parasitol. 210, 107831. 10.1016/j.exppara.2020.107831 31926147

[B71] Lin H. Zhou J. Lin K. Wang H. Liang Z. Ren X.. (2016). Efficacy of *Scutellaria baicalensis* for the Treatment of Hand, Foot, and Mouth Disease Associated with Encephalitis in Patients Infected with EV71: A Multicenter. Retrospect. Analysis BioMed. Res. Int. 2016, 5697571. 10.1155/2016/5697571 PMC5093290 27840828

[B72] Lin B. C. Harris D. R. Kirkman L. M. D. Perez A. M. Qian Y. W. Schermerhorn J. T.. (2017). FIKK Kinase, a Ser/Thr Kinase Important to Malaria Parasites, Is Inhibited by Tyrosine Kinase Inhibitors. ACS Omega 2, 6605–6612. 10.1021/acsomega.7b00997 30023525 PMC6044879

[B73] Lisgarten J. N. Coll M. Portugal J. Wright C. W. Aymami J. (2002). The antimalarial and cytotoxic drug cryptolepine intercalates into DNA at cytosine-cytosine sites. Nat. Struct. Biol. 9, 57–60. 10.1038/nsb729 11731803

[B74] Liu W. Liu Y. (2016). Youyou Tu: significance of winning the 2015 Nobel Prize in Physiology or Medicine. Cardiovasc. Diagn. Ther. 6, 1–2. 10.3978/j.issn.2223-3652.2015.12.11 26885485 PMC4731589

[B75] Lu Y. Joerger R. Wu C. (2011). Study of the chemical composition and antimicrobial activities of ethanolic extracts from roots of *Scutellaria baicalensis* Georgi. J. Agric. Food Chem. 59, 10934–10942. 10.1021/jf202741x 21866919

[B76] Luca S. V. Macovei I. Bujor A. Miron A. Skalicka-Wozniak K. Aprotosoaie A. C.. (2020). Bioactivity of dietary polyphenols: The role of metabolites. Crit. Rev. Food Sci. Nutr. 60, 626–659. 10.1080/10408398.2018.1546669 30614249

[B77] Lux J. Z. Weiss D. Linden J. V. Kessler D. Herwaldt B. L. Wong S. J.. (2003). Transfusion-associated babesiosis after heart transplant. Emerg. Infect. Dis. 9, 116–119. 10.3201/eid0901.020149 12533293 PMC2873739

[B78] Magnarelli L. A. Dumler J. S. Anderson J. F. Johnson R. C. Fikrig E. (1995). Coexistence of antibodies to tick-borne pathogens of babesiosis, ehrlichiosis, and Lyme borreliosis in human sera. J. Clin. Microbiol. 33, 3054–3057. 10.1128/JCM.33.11.3054-3057.1995 8576376 PMC228637

[B79] Manga H. M. Brkic D. Marie D. E. Quetin-Leclercq J. (2004). In vivo anti-inflammatory activity of *Alchornea cordifolia* (Schumach. Thonn.) Mull. Arg. (Euphorbiaceae). J. Ethnopharmacol. 92, 209–214. 10.1016/j.jep.2004.02.019 15138002

[B80] Matsuu A. Yamasaki M. Xuan X. Ikadai H. Hikasa Y. (2008). In vitro evaluation of the growth inhibitory activities of 15 drugs against *Babesia gibsoni* (Aomori strain). Vet. Parasitol. 157, 1–8. 10.1016/j.vetpar.2008.07.023 18771856

[B81] Mazuz M. L. Golenser J. Fish L. Haynes R. K. Wollkomirsky R. Leibovich B.. (2013). Artemisone inhibits in vitro and in vivo propagation of *Babesia bovis* and *B*. *bigemina* parasites. Exp. Parasitol. 135, 690–694. 10.1016/j.exppara.2013.10.006 24184077

[B82] Mensah K. B. Benneh C. Forkuo A. D. Ansah C. (2019). Cryptolepine, the Main Alkaloid of the Antimalarial *Cryptolepis sanguinolenta* (Lindl.) Schlechter, Induces Malformations in Zebrafish Embryos. Biochem. Res. Int. 2019, 7076986. 10.1155/2019/7076986 31360547 PMC6644280

[B83] Meshnick S. R. Taylor T. E. Kamchonwongpaisan S. (1996). Artemisinin and the antimalarial endoperoxides: from herbal remedy to targeted chemotherapy. Microbiol. Rev. 60, 301–315. 10.1128/MR.60.2.301-315.1996 8801435 PMC239445

[B84] Mesia G. K. Tona G. L. Nanga T. H. Cimanga R. K. Apers S. Cos P.. (2008). Antiprotozoal and cytotoxic screening of 45 plant extracts from Democratic Republic of Congo. J. Ethnopharmacol. 115, 409–415. 10.1016/j.jep.2007.10.028 18068320

[B85] Mills-Robertson F. C. Tay S. C. Duker-Eshun G. Walana W. Badu K. (2012). In vitro antimicrobial activity of ethanolic fractions of *Cryptolepis sanguinolenta* . Ann. Clin. Microbiol. Antimicrob. 11, 16. 10.1186/1476-0711-11-16 22709723 PMC3473295

[B86] Moro M. H. David C. S. Magera J. M. Wettstein P. J. Barthold S. W. Persing D. H. (1998). Differential effects of infection with a Babesia-like piroplasm, WA1, in inbred mice. Infect. Immun. 66, 492–498. 10.1128/IAI.66.2.492-498.1998 9453601 PMC107933

[B87] Munyangi J. Cornet-Vernet L. Idumbo M. Lu C. Lutgen P. Perronne C.. (2018). Effect of *Artemisia annua* and *Artemisia afra* tea infusions on schistosomiasis in a large clinical trial. Phytomedicine 51, 233–240. 10.1016/j.phymed.2018.10.014 30466622 PMC6990975

[B88] Munyangi J. Cornet-Vernet L. Idumbo M. Lu C. Lutgen P. Perronne C.. (2019). Artemisia annua and Artemisia afra tea infusions vs. artesunate-amodiaquine (ASAQ) in treating *Plasmodium falciparum* malaria in a large scale, double blind, randomized clinical trial. Phytomedicine 57, 49–56. 10.1016/j.phymed.2018.12.002 30668322 PMC6990969

[B89] Mustofa A. Benoit-Vical F. Pelissier Y. Kone-Bamba D. Mallie M. (2000). Antiplasmodial activity of plant extracts used-in west African traditional medicine. J. Ethnopharmacol. 73, 145–151. 10.1016/S0378-8741(00)00296-8 11025150

[B90] Nagai A. Yokoyama N. Matsuo T. Bork S. Hirata H. Xuan X.. (2003). Growth-inhibitory effects of artesunate, pyrimethamine, and pamaquine against *Babesia equi* and *Babesia caballi* in in vitro cultures. Antimicrob. Agents Chemother. 47, 800–803. 10.1128/AAC.47.2.800-803.2003 12543697 PMC151728

[B91] Nehrbass-Stuedli A. Boykin D. Tidwell R. R. Brun R. (2011). Novel diamidines with activity against *Babesia divergens* in vitro and *Babesia microti* in vivo. Antimicrob. Agents Chemother. 55, 3439–3445. 10.1128/AAC.01482-10 21537025 PMC3122389

[B92] Nishioka T. Kawabata J. Aoyama Y. (1998). Baicalein, an alpha-glucosidase inhibitor from *Scutellaria baicalensis* . J. Nat. Prod. 61, 1413–1415. 10.1021/np980163p 9834167

[B93] Olliaro P. L. Haynes R. K. Meunier B. Yuthavong Y. (2001). Possible modes of action of the artemisinin-type compounds. Trends Parasitol. 17, 122–126. 10.1016/S1471-4922(00)01838-9 11286794

[B94] Osafo N. Mensah K. B. Yeboah O. K. (2017). Phytochemical and Pharmacological Review of *Cryptolepis sanguinolenta* (Lindl.) Schlechter. Adv. Pharmacol. Sci. 2017, 3026370. 10.1155/2017/3026370 29750083 PMC5661077

[B95] Pan B. Shi X. Ding T. Liu L. (2019). Unraveling the action mechanism of *Polygonum cuspidatum* by a network pharmacology approach. Am. J. Transl. Res. 11, 6790–6811. 31814888 PMC6895524

[B96] Pang H. Xue W. Shi A. Li M. Li Y. Cao G.. (2016). Multiple-Ascending-Dose Pharmacokinetics and Safety Evaluation of Baicalein Chewable Tablets in Healthy Chinese Volunteers. Clin. Drug Investig. 36, 713–724. 10.1007/s40261-016-0418-7 27352310

[B97] Parveen N. Bhanot P. (2019). *Babesia microt*i-*Borrelia burgdorferi* Coinfection. Pathogens 8 (3), 117. 10.3390/pathogens8030117 PMC6789475 31370180

[B98] Passos C. L. Ferreira C. Soares D. C. Saraiva E. M. (2015). Leishmanicidal Effect of Synthetic trans-Resveratrol Analogs. PloS One 10, e0141778. 10.1371/journal.pone.0141778 26517558 PMC4627731

[B99] Price R. van Vugt M. Phaipun L. Luxemburger C. Simpson J. McGready R.. (1999). Adverse effects in patients with acute falciparum malaria treated with artemisinin derivatives. Am. J. Trop. Med. Hyg. 60, 547–555. 10.4269/ajtmh.1999.60.547 10348227

[B100] Puri B. K. White N. Monro J. A. (2019). The effect of supplementation with *Scutellaria baicalensis* on hepatic function. Med. Hypotheses 133, 109402. 10.1016/j.mehy.2019.109402 31557595

[B101] Raffalli J. Wormser G. P. (2016). Persistence of babesiosis for >2 years in a patient on rituximab for rheumatoid arthritis. Diagn. Microbiol. Infect. Dis. 85, 231–232. 10.1016/j.diagmicrobio.2016.02.016 27036977

[B102] Rasoanaivo P. Wright C. W. Willcox M. L. Gilbert B. (2011). Whole plant extracts versus single compounds for the treatment of malaria: synergy and positive interactions. Malar. J. 10 Suppl 1, S4. 10.1186/1475-2875-10-S1-S4 21411015 PMC3059462

[B103] Restani P. Di Lorenzo C. Garcia-Alvarez A. Badea M. Ceschi A. Egan B.. (2016). Adverse Effects of Plant Food Supplements Self-Reported by Consumers in the PlantLIBRA Survey Involving Six European Countries. PloS One 11, e0150089. 10.1371/journal.pone.0150089 26928206 PMC4771165

[B104] Rizk M. A. El-Sayed S. A. Terkawi M. A. Youssef M. A. El Said el Sel S. Elsayed G.. (2015). Optimization of a Fluorescence-Based Assay for Large-Scale Drug Screening against Babesia and Theileria Parasites. PloS One 10, e0125276. 10.1371/journal.pone.0125276 25915529 PMC4411034

[B105] Rizk M. A. AbouLaila M. El-Sayed S. A. E. Guswanto A. Yokoyama N. Igarashi I. (2018). Inhibitory effects of fluoroquinolone antibiotics on *Babesia divergens* and *Babesia microti*, blood parasites of veterinary and zoonotic importance. Infect. Drug Resist. 11, 1605–1615. 10.2147/IDR.S159519 30310296 PMC6166754

[B106] Rizk M. A. Ji S. W. Liu M. M. El-Sayed S. A. Li Y. C. Byamukama B.. (2020). Closing the empty anti-*Babesia gibsoni* drug pipeline in vitro using fluorescence-based high throughput screening assay. Parasitol. Int. 75, 102054. 10.1016/j.parint.2020.102054 31927139

[B107] Sanchez-Vicente S. Tagliafierro T. Coleman J. L. Benach J. L. Tokarz R. (2019). Polymicrobial Nature of Tick-Borne Diseases. mBio 10 (5), e02055–19. 10.1128/mBio.02055-19 PMC6737246 31506314

[B108] Sawer I. K. Berry M. I. Brown M. W. Ford J. L. (1995). The effect of cryptolepine on the morphology and survival of *Escherichia coli*, *Candida albicans* and *Saccharomyces cerevisiae* . J. Appl. Bacteriol. 79, 314–321. 10.1111/j.1365-2672.1995.tb03143.x 7592125

[B109] Scott J. D. Scott C. M. (2018). Human Babesiosis Caused by *Babesia duncani* Has Widespread Distribution across Canada. Healthcare-Basel 6 (2), 49. 10.3390/healthcare6020049 PMC6023460 29772759

[B110] Shan B. Cai Y. Z. Brooks J. D. Corke H. (2008). Antibacterial properties of *Polygonum cuspidatum* roots and their major bioactive constituents. Food Chem. 109, 530–537. 10.1016/j.foodchem.2007.12.064

[B111] Shin N. R. Gu N. Choi H. S. Kim H. (2020). Combined effects of *Scutellaria baicalensis* with metformin on glucose tolerance of patients with type 2 diabetes via gut microbiota modulation. Am. J. Physiol. Endocrinol. Metab. 318, E52–E61. 10.1152/ajpendo.00221.2019 31770016

[B112] Shoemaker M. Hamilton B. Dairkee S. H. Cohen I. Campbell M. J. (2005). In vitro anticancer activity of twelve Chinese medicinal herbs. Phytother. Res. 19, 649–651. 10.1002/ptr.1702 16161030

[B113] Skinner T. S. Manning L. S. Johnston W. A. Davis T. M. (1996). In vitro stage-specific sensitivity of *Plasmodium falciparum* to quinine and artemisinin drugs. Int. J. Parasitol. 26, 519–525. 10.1016/0020-7519(96)89380-5 8818732

[B114] Smith R. P. Hunfeld K. P. Krause P. J. (2020). Management strategies for human babesiosis. Expert Rev. Anti Infect. Ther. 18, 625–636. 10.1080/14787210.2020.1752193 32268823

[B115] Smol’ianinov E. S. Gol’dberg V. E. Matiash M. G. Ryzhakov V. M. Boldyshev D. A. Litvinenko V. I.. (1997). [Effect of *Scutellaria baicalensis* extract on the immunologic status of patients with lung cancer receiving antineoplastic chemotherapy]. Eksp. Klin. Farmakol. 60, 49–51. 9460600

[B116] Stebbings S. Beattie E. McNamara D. Hunt S. (2016). A pilot randomized, placebo-controlled clinical trial to investigate the efficacy and safety of an extract of *Artemisia annua* administered over 12 weeks, for managing pain, stiffness, and functional limitation associated with osteoarthritis of the hip and knee. Clin. Rheumatol. 35, 1829–1836. 10.1007/s10067-015-3110-z 26631103

[B117] Swei A. O’Connor K. E. Couper L. I. Thekkiniath J. Conrad P. A. Padgett K. A.. (2019). Evidence for transmission of the zoonotic apicomplexan parasite Babesia duncani by the tick *Dermacentor albipictus* . Int. J. Parasitol. 49, 95–103. 10.1016/j.ijpara.2018.07.002 30367862 PMC10016146

[B118] Tagboto S. Townson S. (2001). Antiparasitic properties of medicinal plants and other naturally occurring products. Adv. Parasitol. 50, 199–295. 10.1016/S0065-308X(01)50032-9 11757332

[B119] Tempesta M. S. (2010). The clinical efficacy of *Cryptolepis sanguinolenta* in the treatment of malaria. Ghana Med. J. 44, 1–2. 21326982 PMC2956313

[B120] Tona L. Kambu K. Ngimbi N. Cimanga K. Vlietinck A. J. (1998). Antiamoebic and phytochemical screening of some Congolese medicinal plants. J. Ethnopharmacol. 61, 57–65. 10.1016/S0378-8741(98)00015-4 9687082

[B121] Trinh H. Yoo Y. Won K. H. Ngo H. T. T. Yang J. E. Cho J. G.. (2018). Evaluation of in-vitro antimicrobial activity of Artemisia apiacea H. and *Scutellaria baicalensis* G. extracts. J. Med. Microbiol. 67, 489–495. 10.1099/jmm.0.000709 29504922

[B122] Vannier E. Krause P. J. (2009). Update on babesiosis. Interdiscip. Perspect. Infect. Dis. 2009, 984568. 10.1155/2009/984568 19727410 PMC2734943

[B123] Vannier E. Krause P. J. (2012). Human babesiosis. N. Engl. J. Med. 366, 2397–2407. 10.1056/NEJMra1202018 22716978

[B124] Vannier E. G. Diuk-Wasser M. A. Ben Mamoun C. Krause P. J. (2015). Babesiosis. Infect. Dis. Clin. North Am. 29, 357–370. 10.1016/j.idc.2015.02.008 25999229 PMC4458703

[B125] Virji A. Z. Thekkiniath J. Ma W. X. Lawres L. Knight J. Swei A.. (2019). Insights into the evolution and drug susceptibility of *Babesia duncani* from the sequence of its mitochondrial and apicoplast genomes. Int. J. Parasitol. 49, 105–113. 10.1016/j.ijpara.2018.05.008 30176236 PMC6395566

[B126] W.H.O.W.P.S (2012). Effectiveness of Non-Pharmaceutical Forms of Artemisia annua L. against malaria. Available at: https://www.who.int/malaria/position_statement_herbal_remedy_artemisia_annua_l.pdf (Accessed August 28, 2020).

[B127] Walle T. (2011). Bioavailability of resveratrol. Ann. N. Y. Acad. Sci. 1215, 9–15. 10.1111/j.1749-6632.2010.05842.x 21261636

[B128] Wang J. Zhang C. J. Chia W. N. Loh C. C. Li Z. Lee Y. M.. (2015). Haem-activated promiscuous targeting of artemisinin in *Plasmodium falciparum* . Nat. Commun. 6, 10111. 10.1038/ncomms10111 26694030 PMC4703832

[B129] World Health Organization (WHO) (2015). Guidelines for the Treatment of Malaria 2015 (Geneva, Switzerland: World Health Organization).

[B130] Wright C. W. Phillipson J. D. Awe S. O. Kirby G. C. Warhurst D. C. QuetinLeclercq J.. (1996). Antimalarial activity of cryptolepine and some other anhydronium bases. Phytother. Res. 10, 361–363. 10.1002/(SICI)1099-1573(199606)10:4<361::AID-PTR845>3.0.CO;2-N

[B131] Wu J. Hu D. Wang K. X. (2008). [Study of *Scutellaria baicalensis* and Baicalin against antimicrobial susceptibility of *Helicobacter pylori* strains in vitro]. Zhong Yao Cai 31, 707–710. 18826148

[B132] Yang M. Guo M. Y. Luo Y. Yun M. D. Yan J. Liu T.. (2017). Effect of *Artemisia annua* extract on treating active rheumatoid arthritis: A randomized controlled trial. Chin. J. Integr. Med. 23, 496–503. 10.1007/s11655-016-2650-7 28035541

[B133] Yao J. M. Zhang H. B. Liu C. S. Tao Y. Yin M. (2015). Inhibitory effects of 19 antiprotozoal drugs and antibiotics on *Babesia microti* infection in BALB/c mice. J. Infect. Dev. Ctries 9, 1004–1010. 10.3855/jidc.5500 26409742

[B134] Zhang Y. Bai C. Shi W. Alvarez-Manzo H. Zhang Y. (2020). Identification of Essential Oils Including Garlic Oil and Black Pepper Oil with High Activity against *Babesia duncani* . Pathogens 9 (6), 466. 10.3390/pathogens9060466 PMC7350376 32545549

[B135] Zhao Q. Chen X. Y. Martin C. (2016). *Scutellaria baicalensis*, the golden herb from the garden of Chinese medicinal plants. Sci. Bull. (Beijing) 61 (18), 1391–1398. 10.1007/s11434-016-1136-5 27730005 PMC5031759

[B136] Zhao Q. Luan X. Zheng M. Tian X. H. Zhao J. Zhang W. D.. (2020). Synergistic Mechanisms of Constituents in Herbal Extracts during Intestinal Absorption: Focus on Natural Occurring Nanoparticles. Pharmaceutics 12 (2), 128. 10.3390/pharmaceutics12020128 PMC7076514 32028739

