# Supplementary material for: Botanical Medicines Cryptolepis sanguinolenta, Artemisia annua, Scutellaria baicalensis, Polygonum cuspidatum, and Alchornea cordifolia Demonstrate Inhibitory Activity Against Babesia duncani
Source: Front Cell Infect Microbiol. 2021 Mar 8;11:624745. doi: 10.3389/fcimb.2021.624745 (PMC7982592; doi:10.3389/fcimb.2021.624745)
Supplement: Supplementary file 1 [file Table_1.docx]

**Table S1**. Information on the 46 medicinal herb extracts tested in this study.

| **Product Names** | **Plants** | **Extract parts** | **Extract types** | **Manufacturer** |
| --- | --- | --- | --- | --- |
| Grapefruit seed extract | Citrus x paradisi | Seed | Vegetable glycerin | Nutraceutical Corp |
| Samento | *Uncaria tomentosa* | Bark | Ethanol 20-24% | NutraMedix |
| Cumanda | *Campsiandra angustifolia* | Bark | Ethanol 20-24% | NutraMedix |
| Banderol | *Otoba* sp. | Bark | Ethanol 20-24% | NutraMedix |
| Chuan xin lian | *Andrographis paniculata* | Herb | Vegetable glycerin | Hawaii Pharm |
| Hemp oil | *Cannabis sativa* | Seed | Cold pressed | Zatural Hemp |
| Ban zhi lian | *Scutellaria barbata* | Herb | Vegetable glycerin | Hawaii Pharm |
| Coptis | *Rhizoma coptidis* root | Root | Vegetable glycerin | Hawaii Pharm |
| Uncaria | *Uncaria tomentosa* | Bark | Ethanol 50% | KW Botanicals |
| Black Walnut | *Juglans nigra* fruc | Husk/Hull | Ethanol 45% | KW Botanicals |
| Stevia/Tian ju ye | *Stevia rebaudiana* fol | Herb | Ethanol 25% | KW Botanicals |
| Black walnut (30% EE) | *Juglans nigra* | Husk | Ethanol 30% | Heron Botanicals |
| Black walnut (60% EE) | *Juglans nigra* | Husk | Ethanol 60% | Heron Botanicals |
| Black walnut (90% EE) | *Juglans nigra* | Husk | Ethanol 90% | Heron Botanicals |
| Cryptolepis (30% EE) | *Cryptolepis sanguinolenta* | Root | Ethanol 30% | Heron Botanicals |
| Cryptolepis (60% EE) | *Cryptolepis sanguinolenta* | Root | Ethanol 60% | Heron Botanicals |
| Cryptolepis (90% EE) | *Cryptolepis sanguinolenta* | Root | Ethanol 90% | Heron Botanicals |
| Sweet wormwood (30% EE) | *Artemisia annua* | Herb/aerial parts | Ethanol 30% | Heron Botanicals |
| Sweet wormwood (60% EE) | *Artemisia annua* | Herb/aerial parts | Ethanol 60% | Heron Botanicals |
| Sweet wormwood (90% EE) | *Artemisia annua* | Herb/aerial parts | Ethanol 90% | Heron Botanicals |
| Gou teng | *Uncaria rhynchophylla* | Stalk | Vegetable glycerin | Hawaii Pharm |
| Andrographis | *Andrographis paniculata* | Leaf and stem | Cane alcohol 61-71% | Montana Farmacy |
| Teasel/Gao liang jiang | *Dipsacus fullonum + asper* | Root | Ethanol 40% | KW Botanicals |
| Ashwagandha (90% EE) | *Withania somnifera* | Root | Ethanol 90% | Heron Botanicals |
| Ashwagandha (60% EE) | *Withania somnifera* | Root | Ethanol 60% | Heron Botanicals |
| Ashwagandha (30% EE) | *Withania somnifera* | Root | Ethanol 30% | Heron Botanicals |
| Andrographis (90% EE) | *Andrographis paniculata* | Herb/aerial parts | Ethanol 90% | Heron Botanicals |
| Andrographis (60% EE) | *Andrographis paniculata* | Herb/aerial parts | Ethanol 60% | Heron Botanicals |
| Andrographis (30% EE) | *Andrographis paniculata* | Herb/aerial parts | Ethanol 30% | Heron Botanicals |
| Chinese Skullcap (30% EE) | *Scutellaria baicalensis* | Root | Ethanol 30% | Heron Botanicals |
| Chinese Skullcap (60% EE) | *Scutellaria baicalensis* | Root | Ethanol 60% | Heron Botanicals |
| Chinese Skullcap (90% EE) | *Scutellaria baicalensis* | Root | Ethanol 90% | Heron Botanicals |
| Japanese knotweed (90% EE) | *Polygonum cuspidatum* | Root | Ethanol 90% | Heron Botanicals |
| Japanese knotweed (60% EE) | *Polygonum cuspidatum* | Root | Ethanol 60% | Heron Botanicals |
| Japanese knotweed (30% EE) | *Polygonum cuspidatum* | Root | Ethanol 30% | Heron Botanicals |
| Alchornea | *Alchornea cordifolia* | Leaf | Cane alcohol 61-71% | Montana Farmacy |
| Cistus | *Cistus incanus* (aerial parts) | Aerial parts | Organic ethanol 45% | BioPure Healing Products |
| Licorice | *Glycyrrhiza* spp. root | Root | Vegetable glycerin | Nature’s Answer |
| Echinacea | *Echinacea purpurea* & *Echinacea angustifolia* | *Echinacea purpurea* (whole herb) & *Echinacea angustifolia* (root) | Vegetable glycerin | Horbaach Manufacturing |
| Usnea | *Usnea* spp. | Lichen | Cane alcohol 90-100% | Herb Pharm |
| Eleuthero | *Eleutherococcus senticosus* | Root | Cane alcohol 32-42% & Vegetable glycerin | Herb Pharm |
| Houttuynia | *Houttuynia* | Leaf | Ethanol 20-24% | NutraMedix |
| Bidens | *Bidens pilosa* | Leaf | Cane alcohol 61-71% | Montana Farmacy |
| Barberry | *Berberis vulgaris* | Root | Vegetable glycerin | Hawaii Pharm |
| Reishi | *Ganoderma linghzi* | Fruiting Body | Alcohol 30% | North Spore |
| Osha | *Ligusticum porter* | Root | Vegetable glycerin | Hawaii Pharm |
